# Peptide-Modified Lipid Nanoparticles Boost the Antitumor Efficacy of RNA Therapeutics

**DOI:** 10.1021/acsnano.4c14625

**Published:** 2025-04-03

**Authors:** Gangyin Zhao, Ye Zeng, Wanli Cheng, Sofia Karkampouna, Panagiota Papadopoulou, Bochuan Hu, Shuya Zang, Emma Wezenberg, Gabriel Forn-Cuní, Bruno Lopes-Bastos, Marianna Kruithof-de Julio, Alexander Kros, B. Ewa Snaar-Jagalska

**Affiliations:** † Department of Cellular Tumor Biology, Leiden Institute of Biology, Leiden University, Einsteinweg 55, Leiden 2333 CC, the Netherlands; ‡ Department of Supramolecular & Biomaterials Chemistry, Leiden Institute of Chemistry, Leiden University, Einsteinweg 55, Leiden 2333 CC, the Netherlands; § Urology Research Laboratory, Department for BioMedical Research, University of Bern, Bern 3010, Switzerland; ∥ Department of Urology, Inselspital, Bern University Hospital, University of Bern, Bern 3010, Switzerland; ⊥ Shenzhen Institute of Advanced Technology, Chinese Academy of Sciences, Shenzhen 51800, China

**Keywords:** lipid nanoparticles, CD44, YAP/TAZ siRNA, tumor targeting, zebrafish, patient-derived PDX

## Abstract

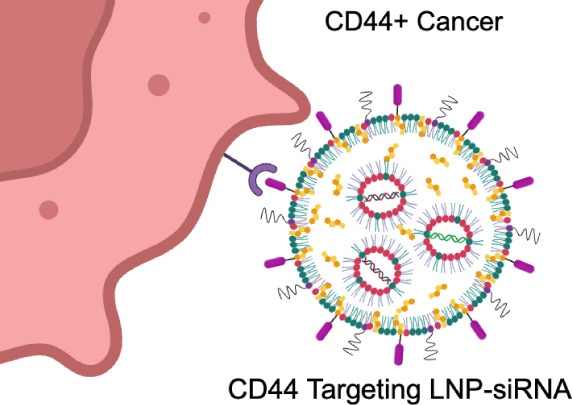

RNA therapeutics offer a promising approach to cancer treatment by precisely regulating cancer-related genes. While lipid nanoparticles (LNPs) are currently the most advanced nonviral clinically approved vectors for RNA therapeutics, their antitumor efficacy is limited by their unspecific hepatic accumulation after systemic administration. Thus, there is an urgent need to enhance the delivery efficiency of LNPs to target tumor-residing tissues. Here, we conjugated the cluster of differentiation 44 (CD44)-specific targeting peptide A6 (KPSSPPEE) to the cholesterol of LNPs via PEG, named AKPC-LNP, enabling specific tumor delivery. This modification significantly improved delivery to breast cancer cells both *in vitro* and *in vivo*, as shown by flow cytometry and confocal microscopy. We further used AKPC-siYT to codeliver siRNAs targeting the transcriptional coactivators YAP and TAZ, achieving potent gene silencing and increased cell death in both 2D cultures and 3D tumor spheroids, outperforming unmodified LNPs. In a breast tumor cell xenografted zebrafish model, systemically administered AKPC-siYT induced robust silencing of YAP/TAZ and downstream genes and significantly enhanced tumor suppression compared to unmodified LNPs. Additionally, AKPC-siYT effectively reduced proliferation in prostate cancer organoids and tumor growth in a patient-derived xenograft (PDX) model. Overall, we developed highly efficient AKPC-LNPs carrying RNA therapeutics for targeted cancer therapy.

## 1 Introduction

Breast cancer (BC) is among the major causes of cancer-related death and is the most common cancer found in women.^1^ Of all patients with BC, 10–15% have aggressive disease, leading to tumor spread to other organs within 3 years of developing the primary tumor.^2^ Patients with metastatic BC have a 5-year survival rate 22%.^3^ Cancer therapy using molecularly targeted small-molecule inhibitors and immunotherapy has improved patients’ quality of life and life expectancy.^4−6^ However, despite these advances, conventional therapies for most cancers often result in severe toxicity, high recurrence rates, and drug resistance.^4,7^ Therefore, novel cancer therapeutics that can provide superior specificity and low toxicity are in pressing need. In this context, gene-targeted therapies are emerging as the next revolution in cancer therapeutics, with RNA therapeutics and gene editing being at the forefront, which allows for the precise targeting of cancer cells while minimizing harm to normal tissues.^8,9^

RNA interference is a naturally occurring, sequence-specific mechanism that regulates approximately 30% of human gene expression at the post-transcriptional level.^10^ It holds tremendous potential for cancer therapies, as it can silence disease-causing genes, particularly those that have developed resistance to traditional treatments or lack ″druggable″ targets using traditional therapeutics (e.g., small molecules, proteins, or monoclonal antibodies).^11,12^ The manipulation of protein expression mediated by small interfering RNA (siRNA) is triggered by the assembly and activation of the RNA-induced silencing complex (RISC), followed by target recognition and cleavage.^13^ However, siRNA is highly negatively charged, immunogenic, and membrane-impermeable, and the effective and safe delivery of them requires protection and payload from a delivery system that can overcome physiological and biological barriers.^14,15^ To this end, lipid nanoparticles (LNPs) serve as the state-of-the-art nonviral vectors to deliver siRNA.^16−20^ In 2018, the FDA approved the first-ever LNP-based RNA interference therapy, Onpattro (patisiran), which is administered intravenously to treat polyneuropathies resulting from transthyretin-mediated amyloidosis (hereditary transthyretin amyloidosis, hATTR).^21,22^ Inspired by that, LNP-based siRNA delivery to treat broad-spectrum intractable diseases is now of significant interest for the development of the next siRNA formulation.^23^ Studies using siRNA therapeutics have notably advanced toward novel therapies against cancer by targeting the mutations in hundreds of genes, including proto-oncogenes and tumor suppressor genes.^15,24^

YAP and TAZ are two highly related transcriptional regulators of the Hippo pathway (hereafter referred to as YAP/TAZ), and they promote tissue proliferation and organ growth while also essential for cancer initiation and the growth of most solid tumors.^25,26^ YAP/TAZ have been reported to be abnormally expressed in cancer cells and are attributed to cancer stem cell properties, proliferation, chemoresistance, and metastasis.^26−31^ YAP/TAZ plays a central role in BC development and malignancy.^32^ Therefore, targeting YAP/TAZ represents a very promising strategy for BC treatment. At present, numerous research reports have proved that knocking down YAP/TAZ expression by RNA interference can effectively inhibit the proliferation and metastasis of cancer cells.^33−35^

Cluster of Differentiation 44 (CD44) is a family of nonkinase single-span transmembrane glycoproteins encoded by the CD44 gene on chromosome 11 in humans.^36,37^ CD44 is not only involved in many biological processes responsible for maintaining the physiological homeostasis of normal cells, such as cell proliferation, cell differentiation, cell migration, angiogenesis, and the presentation of cytokines, chemokines, and growth factors to their corresponding receptors, but also plays an important role in the pathophysiology of cancers.^38−41^ CD44 expression is highly upregulated in cancers and is putatively considered a cancer stem cell (CSC) marker, making it an essential target to eliminate aggressive cancer cells.^42−44^ As a result, various CD44 targeting moieties have been developed and modified on liposomes/nanoparticles for targeted cancer therapy, including hyaluronic acid, aptamer, and anti-CD44 antibody, which showed varied extent of antitumor efficacy.^45−47^ However, these targeting moieties are often large or have complicated modification procedures to fabricate nanoparticles. Short peptides, on the other hand, are much smaller, derived from proteins, perform biological functions similar to proteins, and are easier to conjugate and characterize. They exhibit numerous advantages, including easy synthesis, facile chemical modification, good stability, and ease of combination with other strategies, giving peptides a significant edge in tumor-targeting therapies.^48−51^ A6 peptide (KPSSPPEE) is a urokinase-derived peptide with a high binding affinity to CD44 and shows effective inhibition of the growth and metastasis of CD44-overexpressing tumors.^52−55^ Moreover, A6 exhibits an exceptional safety profile after subcutaneous administration.^56^ Thus, A6 could serve as a superior specific binding moiety to target tumors with CD44 overexpression.

**Scheme 1:**
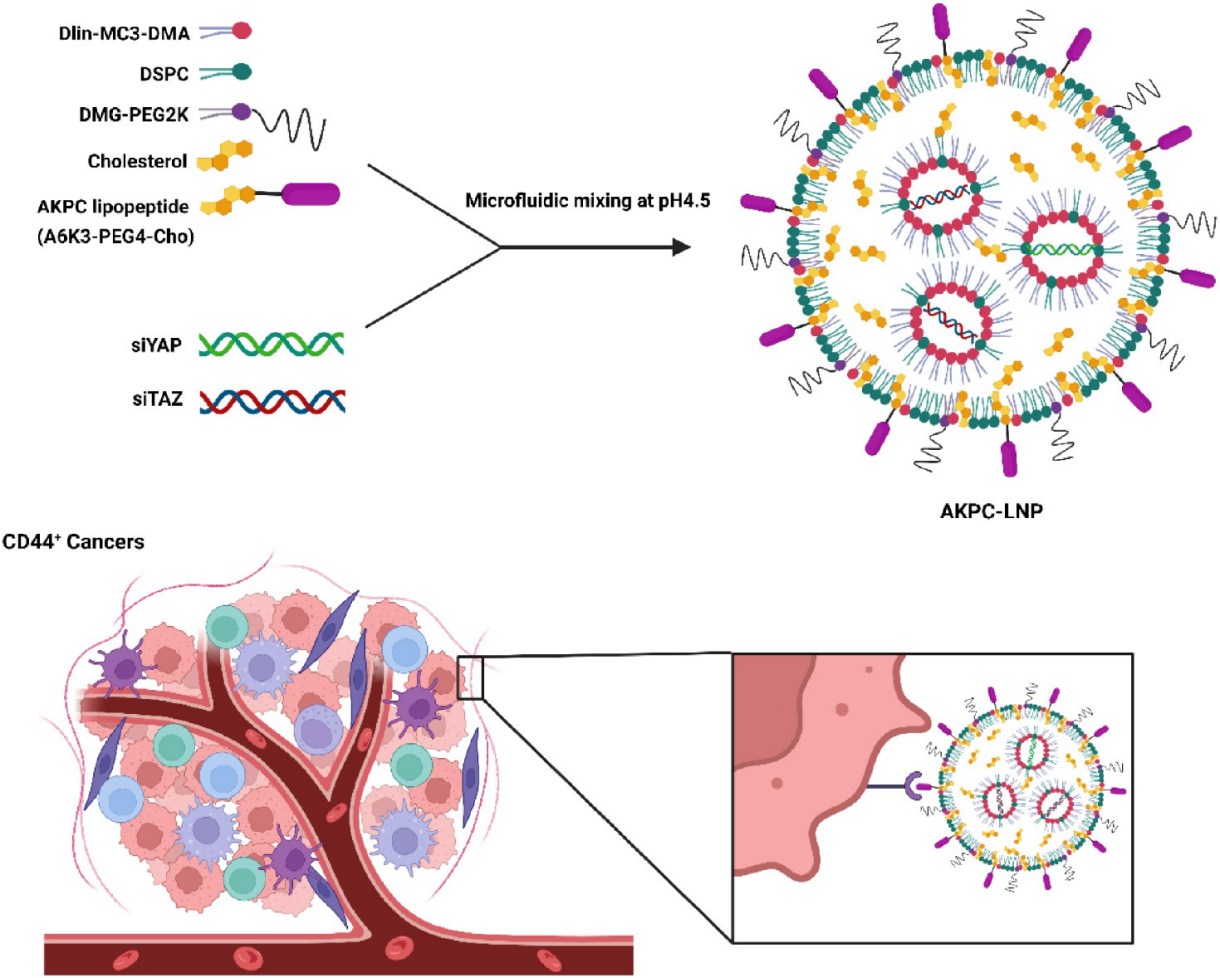
Schematic Representation of Therapeutic Gene Silencing Using LNPs Modified with CD44 Targeted Peptides for YAP/TAZ-siRNA Delivery to CD44^+^ Cancer Cells

Here, we report for the first time that LNPs modified with a CD44-specific peptide achieved enhanced targetability and antitumor efficacy toward human CD44^+^ cancers after codelivering YAP/TAZ-siRNA (Scheme 1). Our CD44-targeting A6 peptide was conjugated to cholesterol-PEG4 on the surface of LNPs through a GGGKKKGK linker, thereby creating a tumor-specific delivery system that we refer to as AKPC-LNP. The AKPC-LNP formulation is engineered to engage with the CD44 receptor protein on the surface of tumor cells via the A6 polypeptide modification on its exterior. This interaction effectively minimizes the spatial separation between the nanoparticles and tumor cells, facilitating the endocytic process. Consequently, this targeted approach enables the efficient delivery of the therapeutic cargo encapsulated within the nanoparticles to the CD44^+^ cells.^57,58^ We evaluated the targeting of breast cancer cells *in vitro* and using xenograft zebrafish as *in vivo* models, which provide a convenient, accurate, visual, and efficient model organism for nanomedicine research.^59,60^ The siRNA has been encapsulated to specifically target the mRNA of YAP/TAZ, a key regulator of tumor cell proliferation and growth. This targeted siRNA is designed to bind to the mRNA, thereby interfering with its translation process and potentially inhibiting the uncontrolled proliferation of tumor cells.^61,62^ After encapsulation of YAP/TAZ-siRNA, we investigated the gene expression, cell apoptosis, and tumor cell growth on 2D cells and 3D spheroids induced by LNPs. Furthermore, we studied the *in vivo* antitumor effect and gene regulation of LNPs on zebrafish xenografts, as well as in prostate cancer (PCa) PDX-derived organoids (PDXO) and PDX models. We anticipate that this general approach could be further employed to enhance disease targeting and the therapeutic efficacy of RNA therapeutics in the future.

## 2 Results

### 2.1 Design of CD44-Specific Peptide-Modified AKPC-LNP

**Figure 1:**
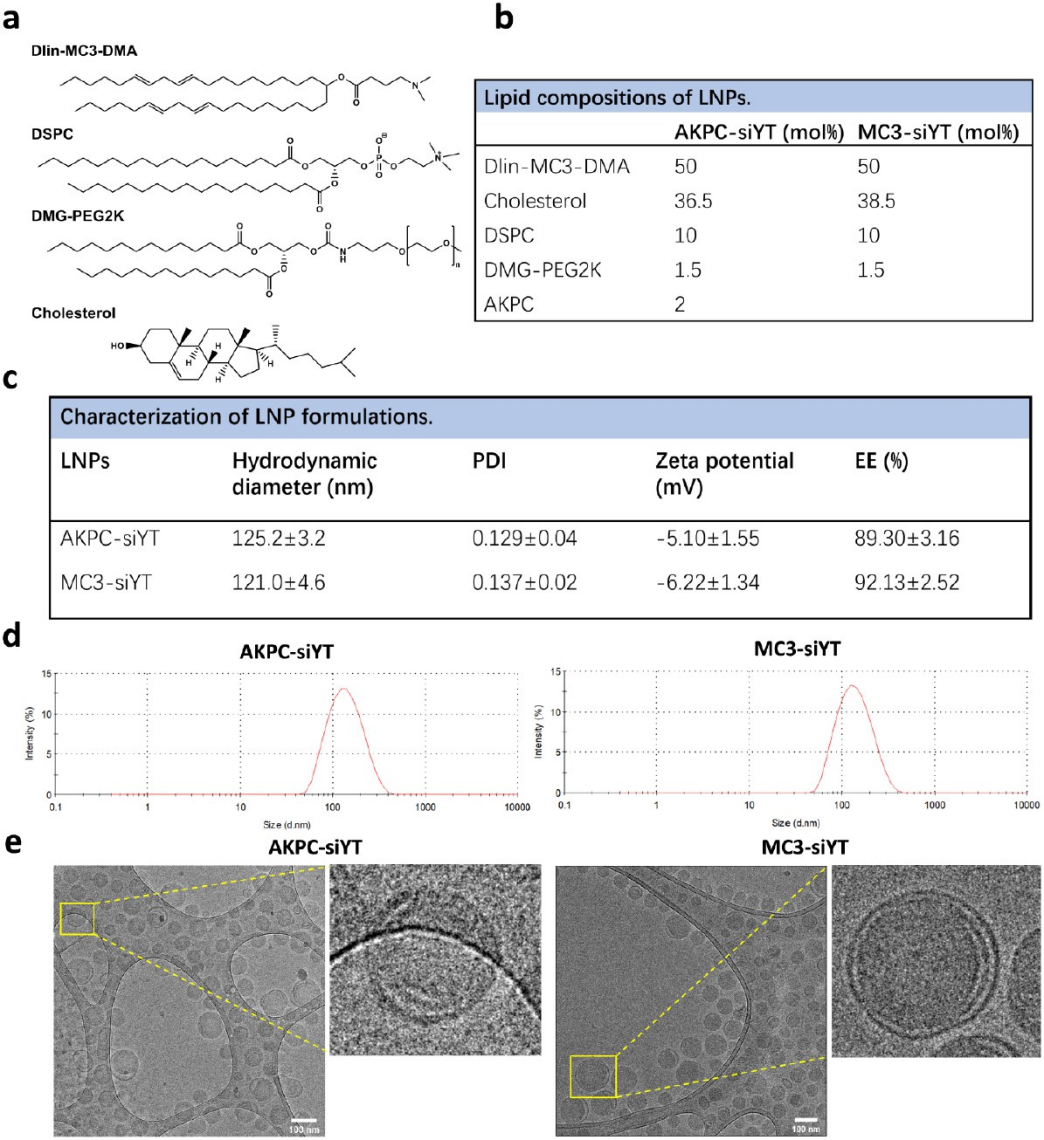
Design and characterization of CD44-specific peptide -modified LNP. **a**, Lipid structures used for the preparation of LNPs. **b**, Lipid compositions of LNPs in molar ratio (mol %). **c**, Characterizations of LNPs. **d**, Representative DLS measurements of LNPs. (e) Cryo-TEM images of LNPs. LNPs were encapsulated with nonsense siRNA. Scale bar: 100 nm.

The Onpattro formulation (MC3-LNP) has been optimized to show potent siRNA delivery efficiency.^21^ It was formulated by mixing the ionizable lipid (6Z,9Z,28Z,31Z)-heptatriaconta-6,9,28,31-tetraen-19-yl 4-(dimethylamino)butanoate (Dlin-MC3-DMA, denoted as MC3, 50 mol %), cholesterol (38.5 mol %), 1,2-distearoyl-*sn*-glycero-3-phosphocholine (DSPC, 10 mol %), and PEGylated lipid (DMG-PEG2K, 1.5 mol %) in ethanol via chaotic mixing with an acidic aqueous phase containing YAP/TAZ-siRNAs targeting the transcriptional coactivators YAP and TAZ (pH 4.5) in a microfluidic device (Figure 1a,b).^56^ The ionizable lipid MC3 can be protonated at pH 4, which can condense acidic siRNA. After dialysis against PBS at pH 7, the LNP becomes neutral. LNPs made of ionizable lipids are slightly negatively charged, consistent with zeta potential, as reported in other literatures.^63^ These four lipids are essential for LNP formation and serve different functions: the ionizable lipid (MC3) enables efficient siRNA encapsulation and endosomal escape for intracellular delivery; the helper phospholipid (DSPC) promotes LNPs formation; cholesterol enhances the stability of the lipid bilayer; and the PEGylated lipid improves colloidal stability and reduces protein absorption.^64−67^

To enhance the therapeutic efficacy and targetability of MC3-siYT, we designed lipid nanoparticles with CD44-specific peptide modification using the short A6 peptide (KPSSPPEE) with an additional GGGKKKGK at the C-terminus, which improves the hydrophilicity and binding affinity of targeting peptides.^68^ The lipopeptide AKPC (Ac-KPSSPPEEGGGKKKGK-PEG4-Cho) (Figure S1a) and the control lipopeptide scramble A6 (Cho-PEG4-SPEKPEPS-NH2) (Figure S1b) were synthesized by conjugating the peptide to a PEG linker and cholesterol, following our previous lipopeptide synthesis protocol.^69−74^

We formulated AKPC-siYT with the same lipid compositions as MC3-siYT, but with the addition of 2 mol % lipopeptide AKPC (Figure 1a,b). The hydrodynamic diameters of both MC3-siYT and AKPC-siYT were determined using dynamic light scattering (DLS), which showed similar sizes of around 120 nm with low polydispersities (both PDI < 0.2). Both LNPs exhibited near-neutral zeta potentials and high siRNA encapsulation efficiencies (Figure 1c). Cryogenic transmission electron microscopy (Cryo-TEM) imaging revealed that both LNP formulations predominately had particles with an electron-dense core, whereas AKPC-siYT also showed some particles with an amorphous and lamellar core structure—the diffused and layered arrangement of lipids—which was similar to unmodified MC3-siYT (Figure 1d). In summary, the addition of 2 mol % peptide AKPC to the MC3-siYT formulation did not alter the physicochemical properties of LNPs; thus, differences in cell uptake and silencing potency can be attributed to the presence of CD44-targeted AKPC (*vide infra*).

### 2.2 Binding Affinity of CD44-Specific Peptide-Modified LNPs to Cancer Cells *In Vitro*

**Figure 2:**
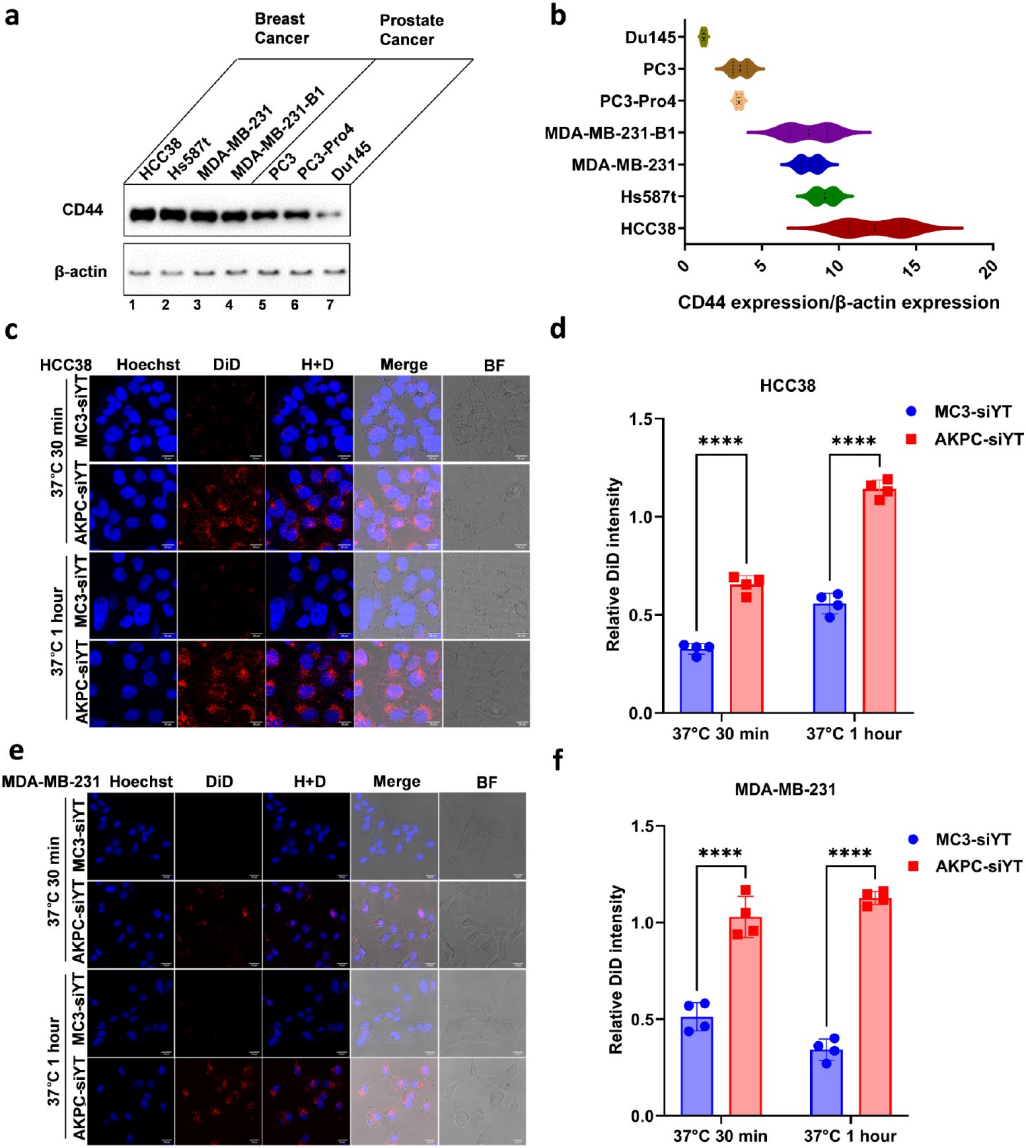
Evaluation of AKPC-siYT targeting breast cancer cells *in vitro*. **a**, Western blot images of CD44 expression in different cell lines. CD44 and β-actin mouse primary antibodies were used to detect protein expression. **b**, Quantification of CD44 expression to β-actin in different cell lines. **c**,**e**, Confocal microscopic images of cellular internalization of LNPs in HCC38 and MDA-MB-231 cells at 37 °C after 30 min and 1 h of incubation. 0.5 mol % DiD was added to the lipids and served as the fluorescent dye. Scale bar represents 20 μm. **d**,**f**, The DiD fluorescence intensity was normalized to Hoechst for the uptake quantification of LNPs by HCC38 and MDA-MB-231 cells. A two-way ANOVA multiple comparison was used to determine the significance of data indicated in d and f (*p < 0.05; **p < 0.01; ***p < 0.001; ****p < 0.0001). In all panels, error bars represent mean ± s.d. (*n* = 3).

To identify suitable cancer cell lines for testing the binding affinity of LNPs modified with AKPC, we measured CD44 expression levels in different breast and prostate tumor cells by Western blotting (WB) (Figure 2a,b). Overall, breast cancer cells expressed much higher levels of CD44 than prostate cancer cells. Therefore, we selected HCC38 and MDA-MB-231 cells for further experiments.

To assess the *in vitro* binding affinity of LNPs to breast cancer cells, we added 0.5 mol % of the far-red fluorescent probe 1,1’-dioctadecyl-3,3,3′,3′-tetramethylindodicarbocyanine,4-chlorobenzenesulfonate salt (DiD) to the lipids and formulated DiD-labeled LNPs. We then used flow cytometry to detect the binding efficiency of LNPs to tumor cells at 4 °C. As expected, CD44-specific lipopeptide-modified AKPC-siYT exhibited notably higher binding affinity than MC3-siYT in both HCC38 and MDA-MB-231 cells (Figure S2a,b).

Next, the cellular uptake efficiency of LNPs at both 37 and 4 °C was evaluated by confocal imaging. Hoechst 33343 was used to stain the nucleus, and quantification was performed by normalizing the DiD fluorescence to the Hoechst fluorescence intensity. In HCC38 cells, AKPC-siYT showed higher cellular uptake efficiency than MC3-siYT after 30 min and 1 h of incubation both at 37 and 4 °C (Figures 2c,d, S2c). Incubation for 1 h induced a significantly higher uptake of both LNPs by HCC38 cells compared to 30 min of incubation. The same enhanced cellular uptake efficiency mediated by AKPC-siYT was also detected in MDA-MB-231 cells compared to MC3-siYT, and both incubation times induced the same effect (Figures 2e,f, S2d).

To further verify whether the high binding affinity of AKPC-siYT to breast cancer cells was CD44 dependent, we generated HCC38 and MDA-MB-231 cell lines with stable CD44 knockdown by using a lentiviral transduction system. WB analysis revealed that both shRNAs targeting sequences significantly reduced the expression of CD44 in HCC38 and MDA-MB-231 cells (Figure S2e). Next, we evaluated the binding affinity of LNPs in these CD44 knockdown breast cancer cells. Interestingly, no difference in cell binding affinity between AKPC-siYT and MC3-siYT was observed in HCC38 and MDA-MB-231 cell lines with the knockdown of CD44 (Figure S2f,g).

Our results show that the efficient uptake of AKPC-siYT by cells *in vitro* is strongly correlated with the targeting of CD44 by the A6 peptide. However, there is also a possible nonspecific binding affinity between the peptide and cells due to the influence of peptide modifications to LNPs. Therefore, we synthesized scrambled A6 peptide-modified LNPs and coincubated them with cells at 4 and 37 °C, respectively. The results showed that scrambled A6-siYT (SA6-siYT) did not increase the uptake of LNPs by cells (Figure S2h–k).

In summary, we demonstrated that LNPs modified with a CD44-specific lipopeptide induced significantly higher cellular uptake efficiency in CD44^+^ HCC38 and MDA-MB-231 cells compared to unmodified MC3-siYT. After the knockdown of CD44 in HCC38 and MDA-MB-231 cells, the high binding selectivity of AKPC-siYT was abolished and became similar to that of unmodified MC3-siYT. These validated that LNPs modified with a CD44-specific peptide mediate highly efficient targeting to CD44^+^ breast cancer cells.

### 2.3 Tumor Targeting Evaluation of CD44-Specific Peptide-Modified LNPs *In Vivo*

After demonstrating that AKPC-siYT specifically targeted breast cancer cells *in vitro*, we investigated the biodistribution of LNPs and their binding to tumor cells *in vivo*. We chose zebrafish as our animal model for *in vivo* evaluation due to their fast growth cycle, economic benefits, transparency, and visibility.^75−78^

First, we examined the biodistribution of DiD-labeled LNPs 1 h after intravenous (IV) injection into the duct of Cuvier (DoC) of 2-days-postfertilization (dpf) Tg(*fli1*:EGFP) in a Casper background fish (green fluorescent blood vessel reporter/Casper fish lines without pigment, further referred to as *fli1*/Casper) into 2-days-postfertilization (dpf) zebrafish embryos. Images of whole zebrafish embryos (10X) revealed that MC3-siYT and AKPC-siYT (in blue) entered the systemic vasculature of zebrafish with free blood circulation. Local high-resolution images (40×) showed that both LNPs were distributed evenly in the tail blood vessels. Additionally, AKPC-siYT and MC3-siYT disseminated through the brain’s blood vessels and even penetrated into the brain cavity (Figure S3a,b). Zoomed-in images of blood vessels in the tail and brain demonstrated that LNPs did not colocate vascular endothelial cells (Figure S3c,d).

**Figure 3:**
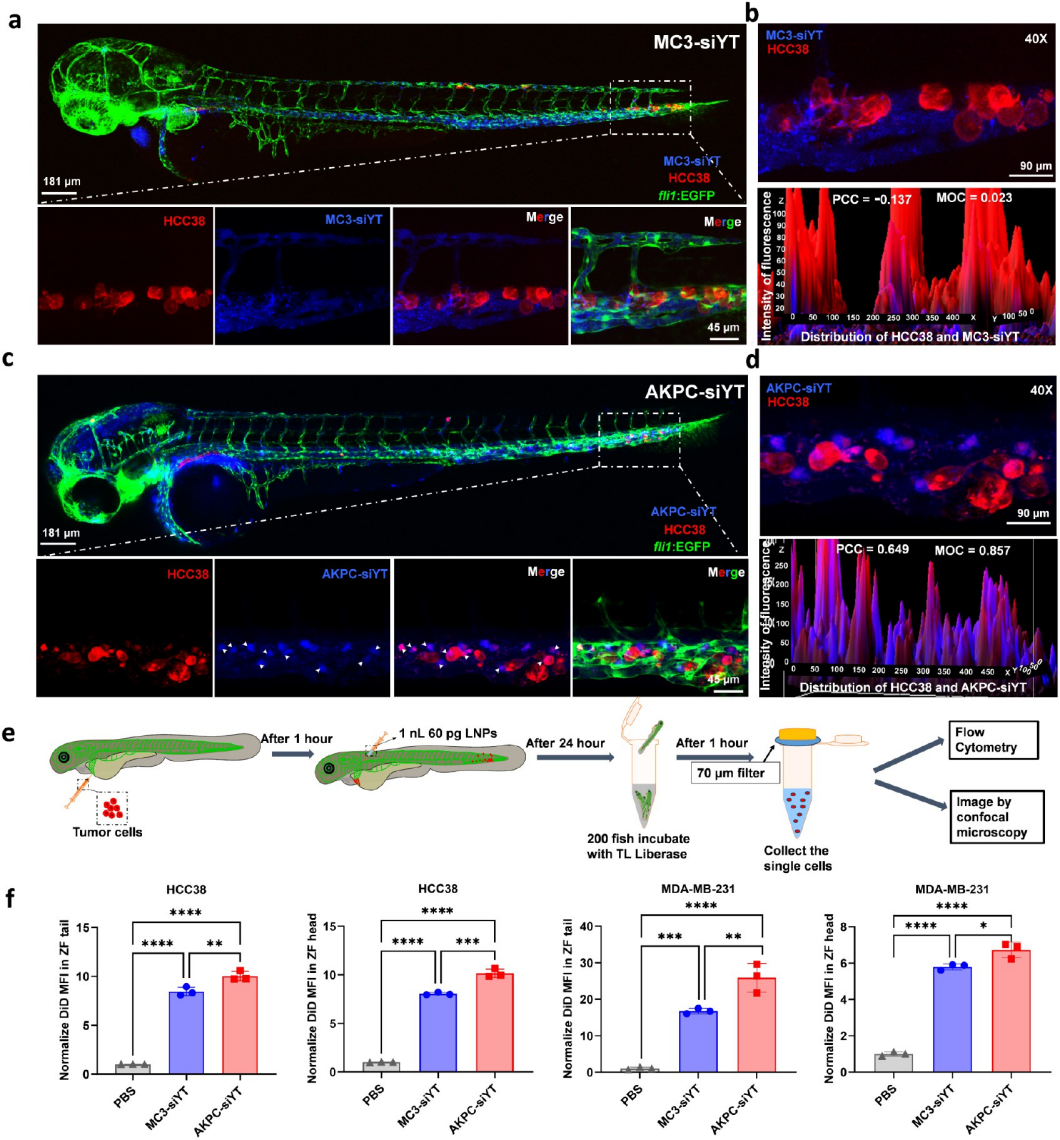
*In vivo* tumor targeting of LNPs to HCC38 cells in zebrafish. **a**,**c**, HCC38 cells (in red), stably expressing mCherry, were implanted into the circulation of 2 dpf *fli1*/EGFP (in green) zebrafish. One hour later, 0.2 mol % DiD (far-red fluorescence) labeled LNPs (1 nL, 60 pg siRNA) (in blue) were injected into the circulation of zebrafish by DoC. SP8 Confocal measured the tumor targeting of LNPs to tumor cells in the zebrafish circulatory system 4 h after LNP injection. **b**,**d**, Colocalization of HCC38 and LNPs in the circulation of zebrafish. ImageJ was used to analyze the distribution of LNPs in the cell area, and the cell fluorescence intensity and LNP fluorescence intensity in the cell area were calculated at the same time. PCC (Pearson correlation coefficient): 1 indicates perfect correlation; 0 indicates random distribution; −1 indicates that colocalization is completely excluded. MOC (Manders overlap coefficient): the value can be 0–1, where 1 indicates complete overlap and 0 indicates complete separation.^81−83^**e**, Schematic diagram of the isolation of tumor cells from zebrafish. **f**, Flow cytometry analysis of LNPs uptake in HCC38 and MDA-MB-231 transplanted in the zebrafish tail and head. The DiD mean fluorescence intensity was normalized to the PBS group for the quantification of LNPs uptake by HCC38 and MDA-MB-231 cells. An ordinary one-way ANOVA multiple comparison was used to determine the significance of data indicated in **f** (*p < 0.05; **p < 0.01; ***p < 0.001; ****p < 0.0001). In all panels, error bars represent mean ± s.d. (*n* = 3).

Next, we tested the targetability of LNPs to tumor cells (HCC38 and MDA-MB-231) xenografted into zebrafish *in vivo*. To do this, breast cancer cells expressing mCherry (red fluorescent protein) were injected into DoC of *fli1*/Casper fish at 2 dpf, as previously established.^79^ The xenograft cells homogeneously disseminated through the circulatory system to the tail of the zebrafish, where they attached to endothelial cells of the vessels and formed multiple tumor foci.^77,79^ One hour postinjection (hpi) of the tumor cells, far-red fluorescent DiD-labeled LNPs were injected through the dorsal aorta into zebrafish (Figure S4a). Four hours later, confocal microscope imaging of entire embryos and high-resolution imaging of the caudal hematopoietic tissue (CHT) revealed that unmodified MC3-siYT did not specifically target HCC38 and MDA-MB-231 cells in embryos at the CHT (Figures 3a and S4b). In particular, colocalization analysis of locally enlarged high-resolution images showed no colocalization of MC3-siYT and tumor cells in the CHT site of zebrafish, as evidenced by Pearson’s correlation coefficient (PCC) and Mander’s Overlap Coefficient (MOC) calculations (Figures 3b and S4c). In contrast, CD44 peptide-modified AKPC-siYT showed specific targeting to both engrafted breast cancer cells, as observed by the appearance of a color change (Figures 3c and S4d). The spatial distribution of MC3-siYT and tumor cell fluorescence intensity in 3D images of zebrafish CHT demonstrated that MC3-siYT showed no colocalization with HCC38 and MDA-MB-231, while the fluorescence intensity of AKPC-siYT and tumor cells displayed clear colocalization in three dimensions (Figures 3d and S4e). The colocalization calculation of PCC and MOC further confirmed that AKPC-siYT (>0.5) induced strong colocalization with tumor cells, while MC3-siYT (near 0.0) showed no colocalization with tumor cells. Combined with PCC and MOC calculations, these results prove that AKPC-siYT specifically targets HCC38 and MDA-MB-231 cells in the zebrafish tumor model.

### 2.4 Tumor Targeting of CD44-Specific Peptide-Modified LNPs in an Orthotopically Transplanted Model

In addition to injection into the DoC, tumor cells can also be implanted into the hindbrain of zebrafish embryos, where they can grow in situ as an orthotopically transplanted model.^80^ With this, we set out to investigate whether an LNP modified with CD44-specific peptides could target tumor cells that remained at the original site without metastasis.

To verify this, breast cancer cells expressing mCherry were implanted into the hindbrain of 2-day-old Tg(*fli1*:EGFP)/Casper zebrafish embryos. DiD-labeled LNPs were then injected into the zebrafish through the dorsal aorta one h after tumor inoculation (Figure S5a). Microscopic images were taken 4 h later and analyzed, as described above, and tumor cells remained in the hindbrain cavity of zebrafish embryos in the course of the experiment. LNPs injected into blood vessels passed through the vascular wall to enter the hindbrain cavity before interacting with tumor cells. Confocal images showed that both MC3-siYT and AKPC-siYT infiltrated the hindbrain of zebrafish from the blood vessels. Notably, AKPC-siYT specifically targeted tumor cells in the hindbrain, while MC3-siYT did not bind to tumor cells (Figures S5b,d and S6a,c). Quantification of the colocalization of LNPs and tumor cells in the 3D structure of the hindbrain by fluorescence intensity distribution, PCC, and MOC values indicated that AKPC-siYT colocalized with tumor cells, while unmodified MC3-siYT showed no colocalization with tumor cells in the hindbrain (Figures S5c,e and S6b,d).

To further validate the tumor-targeting specificity of LNPs in zebrafish, we conducted injections of tumor cells and LNPs into 3 dpf zebrafish. Subsequently, we isolated the tumor cells and examined the uptake of LNPs by these cells *in vivo* using both flow cytometry and confocal microscopy (Figure 3e). High-resolution single-cell imaging revealed that HCC38 and MDA-MB-231 cells exhibited greater internalization of AKPC-siYT compared to MC3-siYT (Figure S7 and Video S1). Quantitative analysis via flow cytometry demonstrated that HCC38 and MDA-MB-231 cells displayed a higher uptake efficiency of AKPC-siYT relative to MC3-siYT in both the brain and tail regions of the zebrafish (Figure 3f).

We also injected scramble A6 peptide-modified SA6-siYT into zebrafish to test the efficiency of the cellular uptake of LNPs *in vivo*. The results showed that neither HCC38 nor MDA-MB-231 had specificity for the uptake of SA6-siYT in zebrafish (Figure S8).

In conclusion, modification of LNPs with the CD44-specific AKPC lipopeptide greatly enhanced the *in vivo* tumor targetability of LNPs to breast cancer cells that had metastasized to the tail and were located *in situ* in the hindbrain.

### 2.5 *In Vitro* Antitumor Evaluation of AKPC-siYT on 2D Cells

YAP/TAZ is a well-known proto-oncogene that regulates the proliferation and division of tumor cells during tumor development.^26^ Studies have shown that YAP/TAZ plays a role in the metastasis of various cancer types, including breast cancer; therefore, inhibiting their expression in breast cancer cells through RNA interference (RNAi) can affect multiple cellular pathways and inhibit tumor development.^84−86^ Here, we investigated whether encapsulating YAP/TAZ siRNAs in AKPC-siYT enhances therapeutic efficacy in CD44^+^ breast cancer cells.

**Figure 4:**
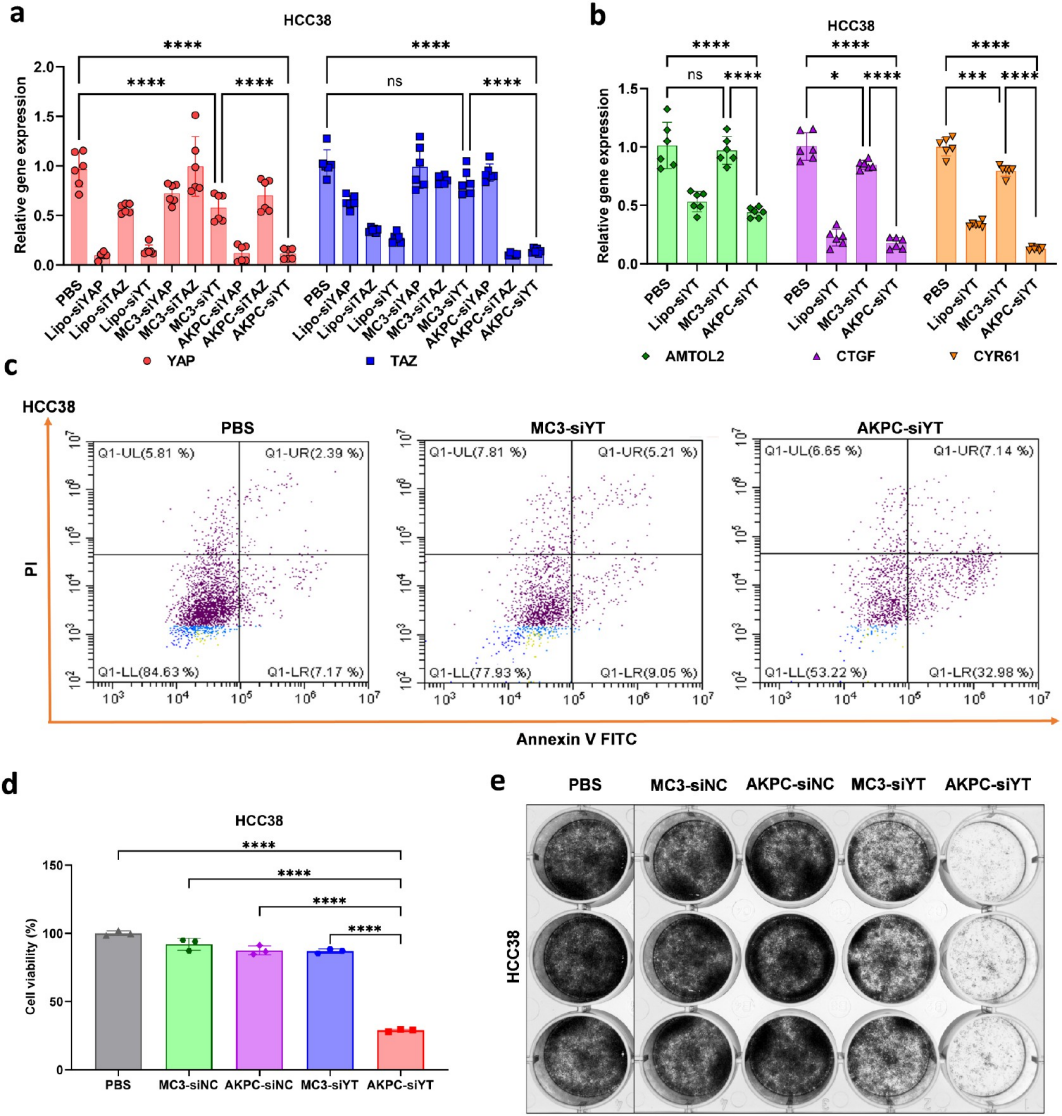
*In vitro* antitumor effects of AKPC-siYT on 2D cells. **a**, RT-PCR results after codelivery of siYAP and siTAZ to HCC38 cells. PBS (negative control); Lipo-siYAP, -siTAZ, -siYT (positive control): lipofectamine containing siYAP, siTAZ, siYAP, and a siTAZ mixture; MC3-siYAP, -siTAZ, -siYT: MC3-LNP containing YAP siRNA, TAZ siRNA, and a YAP and TAZ siRNA mixture; AKPC-siYAP, -siTAZ, -siYT: AKPC-LNPs containing YAP siRNA, TAZ siRNA, a YAP and TAZ siRNA mixture. The concentrations of siRNAs were constant for all conditions (siRNA, 2 μg/mL). **b**, RT-PCR results of the downstream gene after codelivery of siYAP and siTAZ to HCC38 cells. Two-way ANOVA was used to determine the significance of the comparisons of data indicated in **a**, **b** (*p < 0.05; **p < 0.01; ***p < 0.001; ****p < 0.0001). In all panels, error bars represent mean ± s.d. (*n* = 3) **c**, Annexin V/PI staining of HCC38 cells after treatments with PBS and LNPs. **d**, Cell viability measurements by WST-1 in HCC38 cells after treatments with LNPs. Ordinary one-way ANOVA was used to determine the significance of the comparisons of data (*p < 0.05; **p < 0.01; ***p < 0.001; ****p < 0.0001). In all panels, error bars represent mean ± s.d. (*n* = 3). **e**, 1000 cells were seeded as single cells in six-well plate, cultured continuously for 12 days, and treated with LNPs on days 1, 4, and 8 (siRNA, 2 μg/mL). Crystal Violet was used to stain cells and count the number of cell colonies on day 12, indicating the proliferation capacity of the cells.

First, we compared the silencing potency of siRNAs after delivery. We incubated HCC38 and MDA-MB-231 cells for four h with the commercial transfection reagent Lipofectamine, MC3-LNPs and AKPC-LNPs containing siYAP, siTAZ, and siYAP/TAZ (siYT), respectively, and measured the mRNA expression of genes after another 48 h of culturing. The qPCR results demonstrated that Lipofectamine, as a positive control, successfully mediated mRNA silencing of YAP, TAZ, and both after codelivery (Figures 4a and S9a). Compared to MC3-LNPs, AKPC-LNPs exhibited significantly higher silencing potency of YAP, TAZ, and both, indicating that AKPC-LNPs can efficiently deliver siRNA to CD44^+^ breast cancer cells. We also measured the mRNA expression of YAP/TAZ downstream genes AMTOL2, CTGF, and CYR61 in breast cancer cells (Figures 4b and S9b). Consistent with the inhibition of YAP/TAZ expression, AKPC-siYT interfered with the downstream genes more efficiently than MC3-siYT. The knockdown of YAP/TAZ expression and attenuation of downstream gene expression with different treatments indicated that AKPC-LNPs displayed higher siRNA delivery efficiency in breast cancer cells.

Next, we evaluated the effect of YAP/TAZ inhibition by gene silencing on the functional induction of cell apoptosis.^85−87^ The apoptosis rates of HCC38 cells after PBS, MC3-siYT, and AKPC-siYT treatment were 9.56%, 14.26%, and 40.12%, respectively, while those of MDA-MB-231 were 3.9%, 8.35%, and 18.49% (Figures 4c and S9c). AKPC-siYT-mediated YAP/TAZ siRNA delivery induced superior cell apoptosis in breast cancer cells than MC3-siYT.

YAP/TAZ knockdown can effectively inhibit the proliferation and growth of tumor cells.^84,87^ Thus, we tested the cell viability of HCC38 and MDA-MB-231 cells after LNP treatments via the WST-1 assay (Figures 4d and S9d). LNPs containing nonsense siRNA (negative control) showed almost no effect on cell viability. The cell viability of MC3-siYT on HCC38 and MDA-MB-231 was 86% and 93%, respectively. In contrast, AKPC-siYT dramatically reduced the tumor cell viability of HCC38 and MDA-MB-231 to 28% and 33%, respectively.

The specific binding of AKPC to CD44 enabled a high extent of cellular endocytosis of AKPC-siYT, efficiently delivering siRNA into the cells and inducing potent inhibition of tumor proliferation. Meanwhile, MC3-siYT did not show a significant tumor suppression effect. This confirms the importance of AKPC in enhancing the efficiency of LNP-mediated siRNA delivery. In addition, we observed a similar cell inhibition efficiency between MC3-siYT and the negative control group (MC3-siNC) during the short 4 h incubation period. We believe that the inefficient delivery method, due to the non-targeted MC3-LNP, can only deliver a small amount of siRNA into the cells for a certain period of time, thus only slightly interfering with gene expression and cell growth.

To further assess the effect of YAP/TAZ gene silencing on cell proliferation over a long time in culture, we plated 1000 single cells in triplicate in six-well plates and incubated them for 12 days with different treatments. Afterward, we stained them for colony formation using crystal violet (Figures 4e and S9e).^88^ As expected, cells treated with PBS, MC3-siNC (siRNA negative control, MC3-LNP contains the nonsense siRNA), and AKPC- siNC (siRNA negative control, AKPC-LNPs contain the nonsense siRNA) proliferated massively and formed a similar number of colonies. The cells in the MC3-siYT also proliferated to a lesser extent. Importantly, the cells in the AKPC-siYT group hardly proliferated and only formed a few colonies.

In summary, the *in vitro* 2D antitumor effect evaluations indicated that the CD44-target peptide-modified AKPC-siYT achieved a higher silencing potency of YAP/TAZ and led to stronger tumor growth inhibition effects when compared to the unmodified MC3-siYT.

### 2.6 Therapeutic Effect of AKPC-siYT in 3D Tumor Spheroids

**Figure 5:**
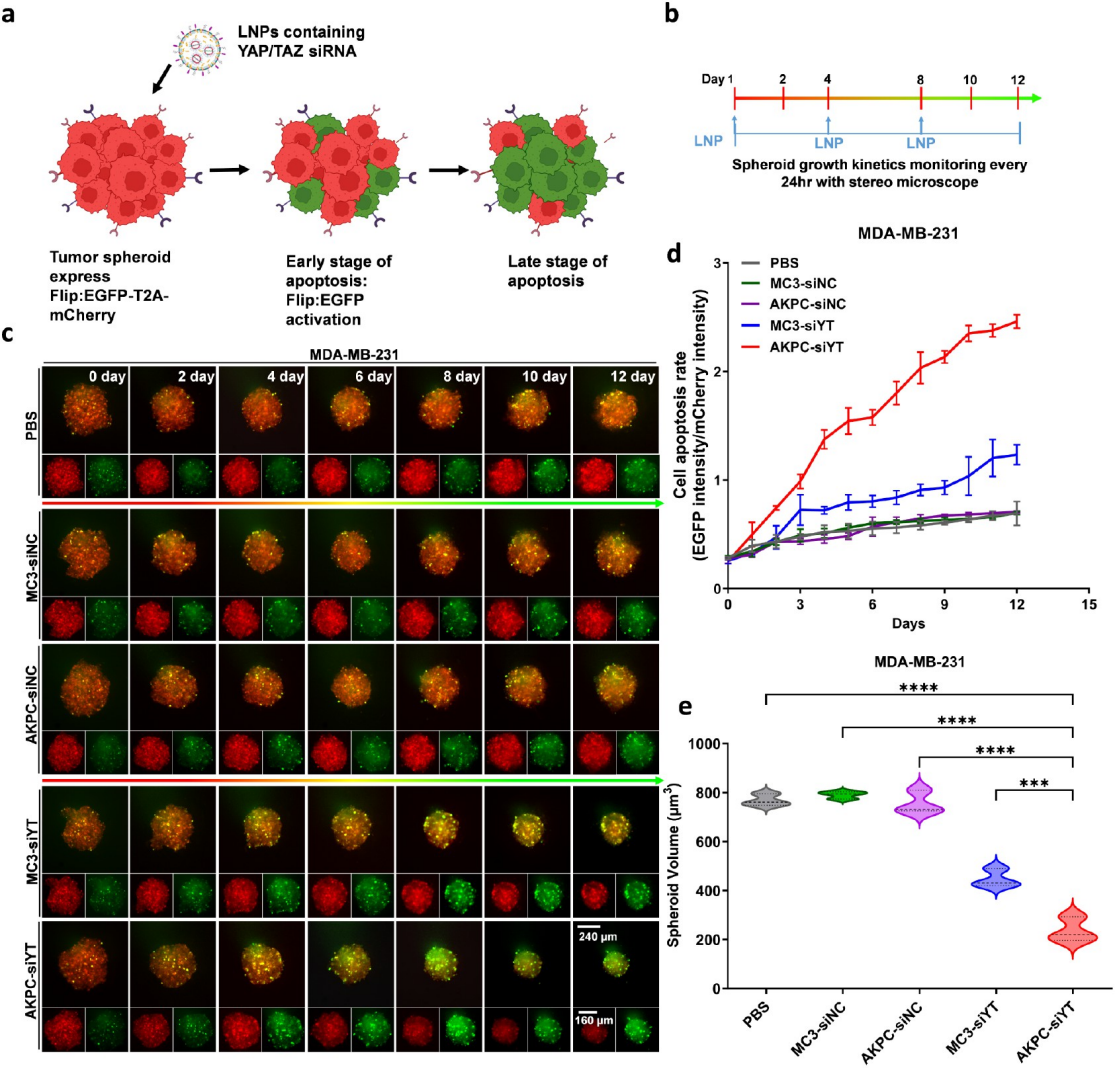
Antitumor effect of AKPC-siYT in 3D tumor spheroids of MDA-MB-231. **a**, Schematic representation of the plasmid containing EGFP-T2A-Caspase3-mCherry (cell apoptosis sensor). **b**, Schematic representation of seeding and treatment of spheroids to monitor spheroid growth kinetics. On days 1, 4, and 8, LNPs were used to treat MDA-MB-231-derived spheroids in different groups (siRNA, 2 μg/mL). A stereo microscope was used to record the tumor spheroids each day for 12 consecutive days. **c**, Images of representative MDA-MB-231 spheroids over time after treatments with LNPs (MC3-siNC/AKPC-siNC: LNPs contain a negative control of siRNA; MC3-siYT/AKPC-siYT: LNPs contain a siYAP and siTAZ mixture), Flip:EGFP intensity (in green) represents cell apoptosis. **d**, Kinetics of the cell apoptosis rate (EGFP intensity/mCherry intensity) from MDA-MB-231 spheroids over time after treatments with LNPs. **e**, MDA-MB-231 spheroids volume calculated by image J on day 12. Ordinary one-way ANOVA was used to determine the significance of the comparisons of data (*p < 0.05; **p < 0.01; ***p < 0.001; ****p < 0.0001). In all panels, error bars represent mean ± s.d. (*n* = 3).

After the 2D *in vitro* antitumor effect evaluation of LNPs loading YAP/TAZ-siRNA, we proceeded to investigate its therapeutic efficacy on 3D tumor spheroids, which better emulate the treatment requirements of a tumor mass.^89−93^ To accurately measure apoptosis in 3D spheroids following gene interference, we introduced an apoptosis reporter system into HCC38 and MDA-MB-231 cells before the generation of 3D tumor spheroids. In this reporter, the fluorescence of GFP is obstructed by DEVD, a conserved cleavage sequence of caspase-3 that can be cleaved upon caspase-3 activation in apoptotic cells, thereby causing GFP to re-emit green fluorescence (Figure S10a).^94^ Moreover, apoptotic GFP is linked to mCherry via the T2A structure, enabling the simultaneous expression of GFP and mCherry in cells. Consequently, under normal conditions, the 3D spheroids exhibit red fluorescence (mCherry), but they turn green (Flip:EGFP) upon apoptosis following treatment. The fluorescence intensity of mCherry represents the protein expression at the background level, while the fluorescence intensity of GFP indicates the degree of apoptosis (Figure 5a).

HCC38 and MDA-MB-231 cells expressing an apoptotic reporter were seeded in a U-bottom low-attachment 96-well plate and exposed to treatments at 1, 4, and 8 days postseeding (Figure 5b).^95^ The apoptosis rate kinetics of tumor spheroids were monitored every day for 12 days using a stereo microscope (Figures 5b,c and S10b). The mean fluorescence intensity of EGFP to mCherry (Flip:EGFP/mCherry) was calculated to illustrate the apoptosis rate (Figures 5d and S10c). When comparing cell apoptosis, we found that mCherry fluorescence remained the same over time, and the Flip:EGFP exhibited little change in the PBS, MC3-siNC, and AKPC-siNC groups. MC3-siYT mediated a certain degree of cell apoptosis with a slight Flip:EGFP fluorescence increase. In contrast, AKPC-siYT induced potent apoptosis of tumor cells with the strongest Flip:EGFP fluorescence increase, which was significantly higher than that of the other groups.

We also analyzed the spheroid volume over time during treatment. Interestingly, the volume of MDA-MB-231 spheroids in all groups decreased slightly from day 1 to day 4, presumably because the cells in the tumor spheroids gradually formed tighter connections from loose connections at day 0 and eventually formed more solid spheroids (Figures 5c and S10b). After day 4, the differences in spheroid volume became evident (Figures 5e and S10d). Spheroids treated with PBS, MC3-siNC, and AKPC-siNC exhibited no tumor volume decrease and remained unchanged from day 6 to day 12. Compared with MC3-siYT, AKPC-siYT treatment significantly decreased the volume of the 3D spheroids. Analysis of the spheroid volume of different groups on day 12 revealed that treatment with MC3-siYT resulted in some spheroid volume decrease (reduced by about 40%) when compared with PBS, MC3-siNC, and AKPC-siNC. Importantly, exposure to AKPC-siYT resulted in the highest reduction of spheroid volume (reduced by about 70%), indicating significant inhibition of tumor growth. For HCC38, we also observed a similar trend in tumor volume after treatments, whereas AKPC-siYT showed the highest tumor spheroid growth inhibition, significantly higher than that of MC3-siYT and other groups.

Overall, the 3D antitumor evaluation showed that, after the CD44-targeting peptide modification, AKPC-siYT was more effective in inducing apoptosis and inhibiting tumor spheroid growth than MC3-siYT.

### 2.7 *In Vivo* Antitumor Effect of AKPC-siYT in Breast Cancer Cells Xenograft Zebrafish Model

*In vitro* 2D and 3D antitumor effect evaluations demonstrated that AKPC-siYT encapsulating YAP/TAZ-siRNA specifically binds to tumor cells with high CD44 expression and efficiently delivers siRNA to these cells, resulting in apoptosis and growth inhibition of tumor cells. To further evaluate the *in vivo* antitumor effect of these LNPs, we used an established zebrafish breast cancer xenograft model.^78^

Before the injection of LNPs *in vivo*, mCherry-expressing HCC38 and MDA-MB-231 cells were implanted into the hindbrain and DoC of 2 dpf *fli1*/Casper zebrafish embryos, respectively. HCC38 cells implanted into the hindbrain via the otic vesicle successfully survived, remaining as single cells in the hindbrain 1 day postinjection, then formed a tumor mass that continued to grow and reached a 3-fold relative tumor burden expansion at 8 dpf (Figure S11a).^80^ When HCC38 cells were injected into the DoC, they homogeneously disseminated to the tail of the zebrafish along with the blood circulation, settled, grew over time, and reached a 4-fold relative tumor burden expansion at 8 dpf (Figure S11b). Similar to HCC38, MDA-MB-231-engrafted zebrafish also induced persistent growth and formed tumor masses in the hindbrain and tail with 2- to 3-fold relative tumor burden expansion at 8 dpf. Therefore, this versatile cancer model provides a sufficient therapeutic window for *in vivo* antitumor evaluation of nanoparticles (Figure S11c,d).

To ensure the safety of LNPs for *in vivo* use, we conducted toxicity tests by injecting varying concentrations of LNPs into zebrafish. We found out that both LNPs showed good biocompatibility at the injected doses (60 pg siRNA) and lower doses; only the higher dose (120 pg siRNA) produced certain toxicity in zebrafish. Overall, the LNPs were deemed safe for *in vivo* injection (Figure S12).

**Figure 6:**
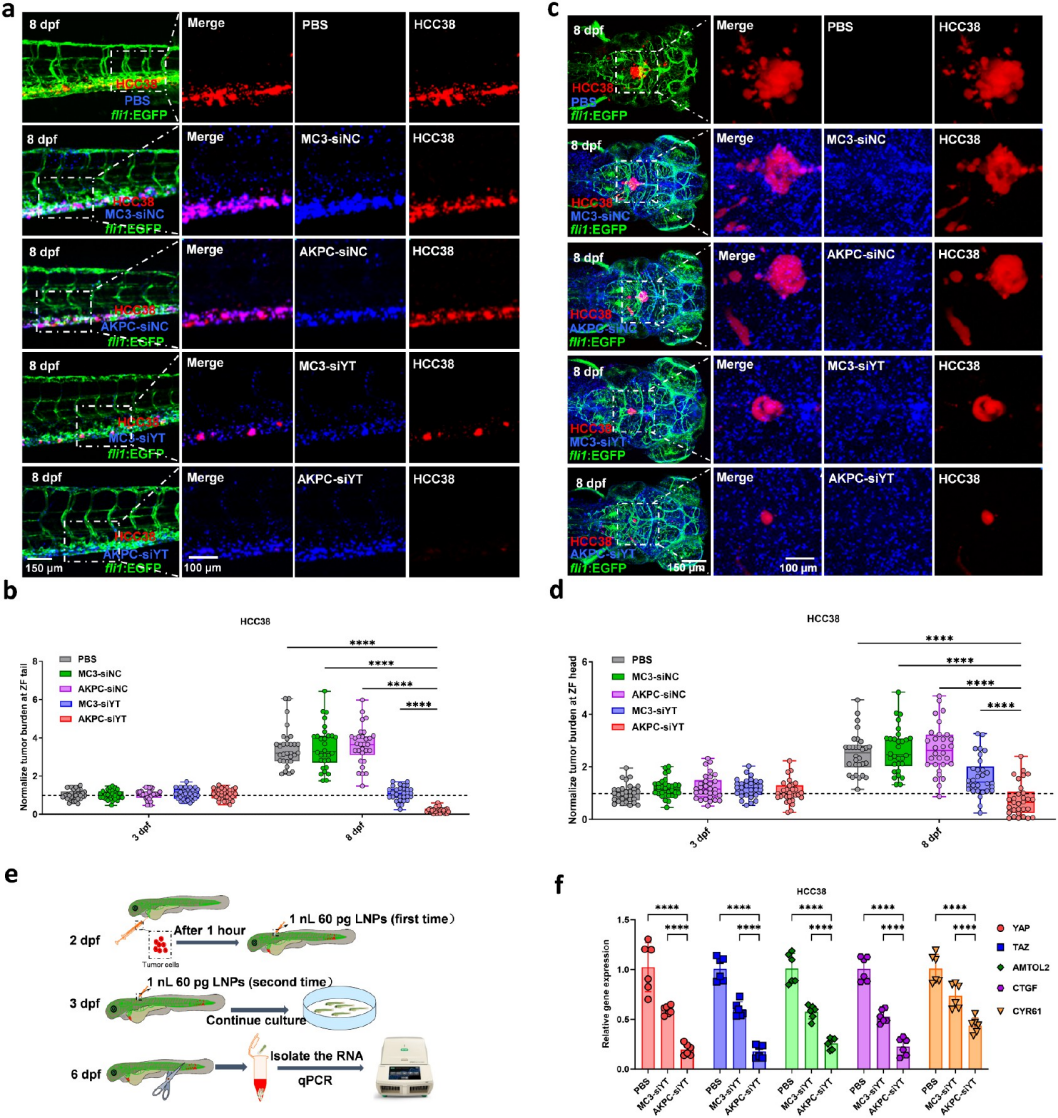
Therapeutic antitumor effect of AKPC-siYT *in vivo* on HCC38 xenografts. **a**, Confocal image of HCC38-mCherry tumor burden (in red) with LNPs-siRNA (in blue) in the circulation of zebrafish at 8 dpf. Green represents vessels of zebrafish embryos. **b**, The relative intensity of red fluorescence (the ratio of fluorescence intensity of each group at 8 dpf to that of the PBS group at 3 dpf) was used to measure tumor burden at CHT sites of zebrafish at 3 dpf and 8 dpf (*n* = 30/group). **c**, Confocal image of HCC38 tumor burden with LNPs-siRNA in the hindbrain at 8 dpf. **d**, The relative intensity of red fluorescence (the ratio of fluorescence intensity of each group at 8 dpf to that of the PBS group at 3 dpf) was used to measure tumor burden in the hindbrain of zebrafish at 3 and 8 dpf (*n* = 30/group). Two-way ANOVA multiple comparisons were used to determine the significance of the comparisons of data indicated in b and d (*p < 0.05; **p < 0.01; ***p < 0.001; ****p < 0.0001). In all panels, error bars represent mean ± s.d. **e**, Schematic representation of RNA isolation of tumor cells from the tail of zebrafish and RT-PCR detection. **f**, RT-PCR results of YAP/TAZ and downstream gene expression in zebrafish after codelivery of siYAP and siTAZ at 8 dpf. Two-way ANOVA was used to determine the significance of the comparisons of data (*p < 0.05; **p < 0.01; ***p < 0.001; ****p < 0.0001). In all panels, error bars represent mean ± s.d. (*n* = 3).

To evaluate the antitumor effect of LNPs on breast cancer cells engrafted into zebrafish, HCC38 and MDA-MB-231 cells were implanted into DoC. Subsequently, DiD-labeled LNPs were injected intravenously into these zebrafish at 1 and 24 h postengraftment of cancer cells, respectively (Figure S13a). At 3 dpf, HCC38 tumor cells disseminated to the tail and settled there, partly forming a cluster (foci) and partly remaining as single cells (Figure S13b). At 8 days postproliferation (dpf), HCC38 in embryos treated with PBS, MC3-siNC, and AKPC-siNC formed tumor clusters and kept growing in the tail (Figure 6a,b). However, treatment with MC3-siYT only partially inhibited tumor growth. In contrast, AKPC-siYT exhibited a significant tumor suppression effect (around 100% inhibition), with almost no tumor cells remaining in the endovascular sites. Tumor volume quantification confirmed that AKPC-siYT induced the highest tumor inhibition compared to all other groups.

In addition to tumor suppression in the tail, we also evaluated tumor inhibition inside the hindbrain, where the tumor was generated *in situ* (Figure S13c). Confocal images at 3 dpf revealed no significant development of solid tumor masses in the hindbrain, and the number of HCC38 cells was consistent across all groups (Figure S13d). At 8 dpf, notable differences were observed in tumor growth among the various groups. While tumor cells in the PBS, MC3-siNC, and AKPC-siNC groups continued to grow and form solid masses, treatment with MC3-siYT showed some degree of tumor inhibition. When compared to MC3-siYT, the AKPC-siYT formulation demonstrates a more pronounced inhibitory effect on the growth of tumor cells within the hindbrain region (Figure 6c,d).

We also verified the antitumor effect of LNPs on MDA-MB-231 cells implanted into the DoC of zebrafish. Again, the same potency of tumor suppression was achieved by AKPC-siYT, which was significantly higher than that of the other groups (Figure S14a–c). Similarly, MDA-MB-231 tumor inhibition inside the hindbrain by LNPs also confirmed that AKPC-siYT induced potent tumor suppression, which was significantly higher than that in the other groups (Figure S15).

To further validate the gene expression of HCC38 and MDA-MB-231 cells after treatment, we cut the tail containing tumor cells of zebrafish treated with MC3-siYT and AKPC-siYT at 8 dpf and extracted the RNA for RT-PCR analysis (Figure 6e). Compared to the control PBS group, the MC3-siYT group showed a reduction of approximately 50% and 40–50% in the expression of YAP/TAZ and its downstream genes, AMTOL2, CYR61, and CTGF, in HCC38 and MDA-MB-231, respectively (Figures 6f and S14d). Notably, AKPC-siYT induced a reduction in the gene expression of YAP/TAZ and its downstream genes by 70–80% and 60–80% in HCC38 and MDA-MB-231, respectively. These results demonstrate that AKPC-siYT induced the highest siRNA delivery efficiency, resulting in strong silencing of YAP, TAZ, and their downstream genes, leading to the inhibition of tumor growth.

In summary, compared with unmodified MC3-siYT, CD44 peptide-modified AKPC-siYT induced enhanced gene silencing and antitumor efficacy in zebrafish breast cancer cell xenografts with a good safety profile.

### 2.8 Antitumor Effect of AKPC-siYT on Prostate Cancer PDX-Derived Organoids and *In Vivo* Tumor Growth

**Figure 7:**
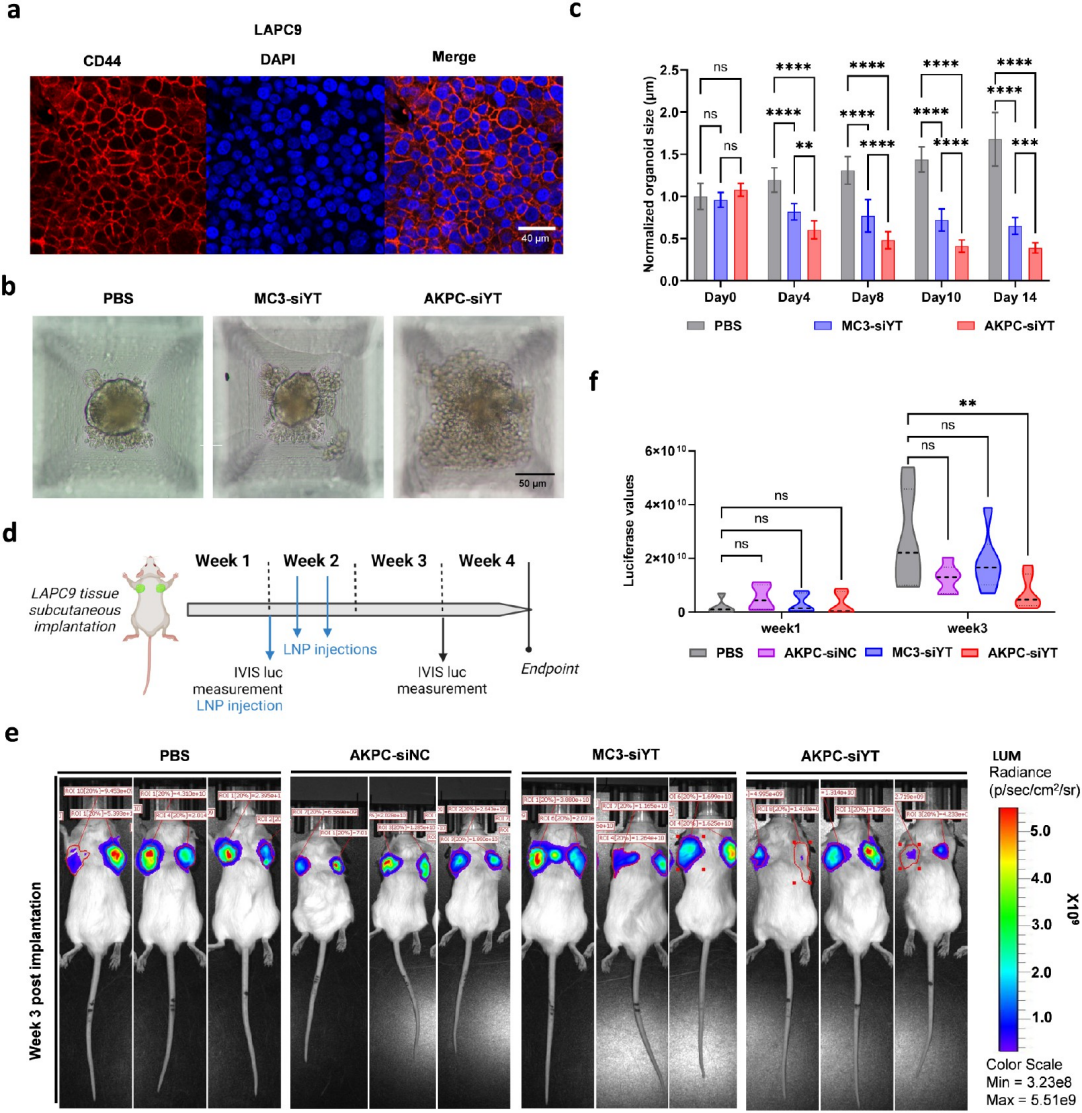
Antitumor effect of AKPC-siYT on prostate cancer PDX-derived organoids and *in vivo* tumor growth. **a**, Prostate cancer PDX model (LAPC9) exhibited high CD44 protein levels (CD44 in red, nuclei marked by DAPI in blue) by immunofluorescence staining. **b**, Morphology of LAPC9 PDX-derived organoids following treatment with PBS, MC3-siYT, and AKPC-siYT (siRNA, 2 μg/mL) for 14 days. Scale bar, 50 μm. **c**, Organoid size of each treatment group (PBS, MC3-siYT, and AKPC-siYT) was measured at different time points (day 0, 4, 8, 10, and 14). Two-way ANOVA multiple comparison was used to determine the significance of the comparisons of data (*p < 0.05; **p < 0.01; ***p < 0.001; ****p < 0.0001). In all panels, error bars represent mean ± s.d. (*n* = 3) **d**, Schematic representation of *in vivo* tumor growth kinetics of LAPC9 PDX following LNP treatment. Bioluminescent LAPC9-copGFP-CBR tumor tissues were subcutaneously (s.c.) implanted in CB17 SCID mice at day 0. Following a lag period of 1 week, mice were subjected to one-week LNP treatment (Days 7, 9, and 11 of week 2). LNPs were sc injected at the tumor-adjacent area (siRNA, 10 μg/tumor, *n* = 3/group). Intravital imaging (IVIS-CT) was used to record tumor dynamics based on stable bioluminescence expression of the LAPC9 tumor cells weekly for 3 consecutive weeks. At the endpoint (week 4), IVIS-CT, tumor collection, and body weight measurement were done. **e**, Bioluminescence images of LAPC9 PDX tumors showing individual tumor areas (*n* = 3/LNP treatment group) at the endpoint. **f,** Violin plot of *in vivo* tumor growth based on average bioluminescence radiance of individual tumors at endpoint (day 28) (*n* = 3 animals/group × 2 tumors). Two-way ANOVA, ŠídÁk’s multiple comparisons test was used to determine the significance of the comparisons of data indicated in d (*p < 0.05; **p < 0.01; ***p < 0.001; ****p < 0.0001). In all panels, error bars represent mean ± s.d.

To validate the antitumor efficacy of LNPs in another tumor type, we employed an advanced androgen-independent bone metastatic PCa patient-derived xenograft tumor model.^96,97^ Similar to breast cancer cells, prostate cancer cells also express high levels of CD44 and play an important role in the growth and metastasis of prostate cancer.^98−100^ The established PDX model (LAPC9) from hormone-resistant and metastatic prostate cancer, characterized by abundant CD44 expression (Figure 7a), was used to further study the tumor-suppressive effect of LNPs. LAPC9 PDX-derived organoids (PDX-Os) were treated *in vitro* with CD44-targeting LNPs to assess cell proliferation over time. To ensure equal starting cell numbers and uniform organoid size, the cells were seeded in micropyramid well SP5D plates and allowed to form 3D organoids for 48 h prior to LNP treatments. Organoid proliferation, indicated by changes in organoid diameter (size), was significantly reduced after treatment with both AKPC-siYT and MC3-siYT compared to the PBS group. In all time points tested (days 4–14), the AKPC-siYT group exhibited consistently smaller organoid sizes than the MC3-siYT group, while the PBS group of PDX-Os showed progressive growth in size (Figure 7b,c).

To determine potential *in vivo* tumor growth effects in response to LNP treatments, bioluminescent LAPC9-copGFP-CBR tumor tissues were subcutaneously implanted into immunodeficient CB17 SCID mice, which were randomized based on bioluminescence and body weight one week postimplantation (Figure 7d). LNPs were locally administered for 1 week (3 times/week during week 2), followed by tumor measurements one week post-treatment (Figure 7d, week 3). Bioluminescence measurements (week 3) demonstrated that the AKPC-siYT group exhibited a lower signal than the MC3-siYT and PBS groups, indicating a reduction in tumor size *in vivo* (Figure 7e,f). At the endpoint (week 4), the tumor size among the treatment groups was similar, suggesting that longer LNP treatment, rather than the short-term (3 administrations) LNP treatment done here, is required for a sustained effect on tumor growth (data not shown). Furthermore, mice after LNP treatments showed a decrease in body weight over time; however, no more than 15% of their original body weight (Figure S16), suggesting tolerance to LNP treatment *in vivo*. In summary, AKPC-siYT treatment effectively targets CD44^+^ cells, leading to a reduction in tumor growth both *in vitro* and *in vivo*.

## 3 Discussion and Conclusion

RNA therapeutics have shown significant progress in the treatment of various pathological diseases by manipulating gene expression or producing therapeutic proteins, including viral infections, cancers, immune diseases, and undruggable genetic disorders.^14^ RNA interference (RNAi)-derived siRNA has emerged as a promising cancer therapeutic since over 85% of genes essential in cancer development are not druggable by traditional drugs.^101^ Additionally, the flexibility of siRNA design allows for the targeted silencing of any gene involved in cancer survival, such as angiogenesis, invasion, immune evasion, drug resistance, and metastasis.^102,103^ Compared to conventional cancer therapy, siRNA performs its function with high potency, tolerance, and specificity.^24,104^ However, due to their natural physiological properties, siRNA requires a suitable delivery platform.^105^ Lipid nanoparticles are currently the state-of-the-art delivery system for different cell types, but their accumulation in the liver and other central organs lowers their efficacy. Therefore, specific targeted lipid nanoparticles can be engineered to improve delivery efficiency and minimize unwanted accumulation in nontargeted organs.

In this study, we developed an efficient nonviral delivery system for siRNA therapeutics using lipid nanoparticles (LNPs) modified with a CD44-specific targeting lipopeptide (AKPC) for cancer therapy. CD44 is a well-known cancer stem cell (CSC) marker, overexpressed in various tumors, including breast, prostate, pancreatic, gastrointestinal, lung, brain, and ovarian cancers.^38,40,106^ We confirmed that the AKPC modification did not alter the physicochemical properties of the LNPs, including size, zeta potential, morphology, and RNA encapsulation efficiency. We demonstrated the *in vitro* tumor-targeting efficacy of the AKPC-LNPs on HCC38 and MDA-MB-231 cells, which are representative triple-negative breast cancer (TNBC) cell lines known for lacking expression of estrogen, progesterone, and ERBB2 receptors and displaying epithelial-to-mesenchymal transition (EMT). TNBC has been recognized as the most malignant breast cancer phenotype with dismal survival.^107,108^ The efficiency of *in vitro* cellular uptake mediated by AKPC-LNPs was superior to that of unmodified MC3-LNPs on both HCC38 and MDA-MB-231 cells. To assess *in vivo* tumor targeting, we used zebrafish models, which are important vertebrate models in developmental biology and cancer research because they develop cancers similar to humans histologically and genetically after mutagen exposure or through transgenesis.^109−111^ We xenografted mCherry-labeled HCC38 and MDA-MB-231 cells onto the DoC and hindbrain of the zebrafish and systemically injected the LNPs, and we observed that CD44 lipopeptide-modified MC3-LNPs specifically targeted the tumor cells, while unmodified MC3-LNPs showed no specific tumor targeting.

Discovering new gene targets for potent antitumor efficacy is crucial in RNA-based cancer therapy. YAP/TAZ, which regulate multiple signaling pathways in cancer cells, are often overexpressed in breast cancer patients and correlate with high histological grade, cancer stem cell enrichment, metastasis, chemoresistance, and poor outcomes.^25,27,112^ Moreover, research has shown that TNBC exhibits higher TAZ mRNA and protein expression than other breast cancer subclasses.^113,114^ After encapsulating siYAP/TAZ in LNPs, we demonstrated that AKPC-siYT was able to silence up to ∼ 85% of YAP/TAZ and ∼15–40% of downstream gene (AMTOL2, CTGF, CYR61) expression *in vitro*, which was significantly higher than naked LNPs. In line with that, AKPC-siYT induced enhanced apoptosis, as evaluated by Annexin V/PI staining, and demonstrated tumor growth inhibition in 2D breast cancer cells. To validate these findings, we further assessed the antitumor effects of AKPC-siYT on 3D spheroids, which have high biological relevance in the tumor microenvironment by replicating many tumor characteristics, including tight junctions, biochemical and mechanical cues from the native extracellular matrix, and gene expression profiles similar to those of xenograft tumors.^89−93^ Additionally, 3D spheroids have displayed the phenotype of cancer stem cells and have contributed to the development and progression of malignancy.^115,116^ By introducing an apoptosis reporter into the cells and establishing 3D tumor spheroids, we observed that AKPC-siYT efficiently activated caspase-3 expression, resulting in a significantly higher apoptosis rate and potent inhibition of 3D spheroid tumor growth

Targeted therapy enhances the therapeutic effect by overcoming the limitations of most nanoparticle delivery systems, whose efficacy is lowered by accumulation in the liver and other central organs. In addition to that, systemically administered targeting LNPs enable efficacious therapy for both localized and disseminated (metastatic and/or hematopoietic) cells.^4,117,118^ In our *in vivo* therapeutic evaluation, we used zebrafish models with tumor cells xenografted in both the DoC and hindbrain (locally and circulating). Specifically, we found that CD44-targeted LNPs induced robust silencing of YAP/TAZ and downstream genes (AMOTL2, CYR61, and CTGF), resulting in significantly enhanced tumor suppression compared to naked MC3-LNPs. In addition, CD44-targeting LNPs resulted in a reduction in organoid size within the prostate cancer model and suppressed tumor growth *in vivo*. A slight body weight decrease was measured (10–15%), with notoxicity observations. Short-term LNP treatment was effective at reducing or delaying tumor growth 1 week after treatment cessation; however, the effect was lost at 2 weeks post-treatment, indicating a requirement for long-term or interval treatment to have a sustained tumor cytotoxic effect.

This targeting strategy we designed in this study, using a CD44-specific targeting lipopeptide to modify lipid nanoparticles, is, to our knowledge, the first example of targeted lipid-nanoparticles for an RNA interface to treat metastatic breast cancers. Our strategy provides a highly flexible, specific, and efficient approach for targeting gene therapy that can be extended to other target sites to enable targeted therapies by changing the targeting moiety in response to tumor-specific cell surface receptors (VEGF, EpCAM, or PSMA), common cell receptors (CD19), and other receptors of transformed cells in diseased tissues.^4,119^ Other alternative approaches have also been used for targeting therapy, such as attaching targeting moieties to the PEGylated lipids or the ionizable lipids, or employing postmodification by conjugating ligands to PEGylated lipids after LNP formulation.^78,117,119,120^ However, the targetability and efficacy might be defaulted or compromised with the above strategies because the plasma-exposed PEGylated lipid desorbs from the LNP surface rapidly in the circulation and transfers to lipoproteins and erythrocytes after administration.^121^ Therefore, our simultaneous targeting modification strategy, by adding targeting moieties directly to other lipids and formulating LNPs, is convenient, straightforward, and capable of scale-up production without losing targeting superiority. Overall, this targeting strategy provides a promising platform for the development of targeted gene therapies for a range of diseases.

In our study, we successfully codelivered siYAP and siTAZ using lipid nanoparticles as a proof-of-concept for a novel cancer therapeutic strategy. This approach can be expanded to deliver other genes, either individually or simultaneously, that are not vital for normal tissues and to silence specific tumor-triggering oncogenes (such as MYC, RAS, and ErbB2). For future applications, we envision the use of targeted lipid nanoparticles as a powerful tool for patient-tailored therapy to silence malfunctioning genes that trigger diseases. Overall, this therapeutic targeted lipid nanoparticle strategy opens new avenues for encapsulating RNA therapeutics as a novel modality for cancer therapy and other diseases as well.

## 4 Methods

### 4.1 Materials

All Fmoc-protected amino acids were purchased from Novabiochem. Piperidine, trifluoroacetic acid, acetonitrile, and dimethylformamide (DMF) were purchased from Biosolve. Dichloromethane (DCM) and ethanol were purchased from Sigma-Aldrich. 1,2-Distearoyl-*sn*-glycero-3-phosphocholine (DSPC) was purchased from Avanti Polar Lipids, DLin-MC3-DMA was purchased from Biorbyt (Cambridge, England), and cholesterol was purchased from Sigma-Aldrich. DiIC_18_(5) solid (1,1’-Dioctadecyl-3,3,3′,3′-Tetramethylindodicarbocyanine, 4-Chlorobenzenesulfonate salt) (DiD) was purchased from Thermo Fisher. Triton X-100 was purchased from Acros Organics. QuantiT RiboGreen RNA reagent and rRNA standards were purchased from Life Technologies. WST-1 reagent was purchased from Sigma-Aldrich. siRNA was purchased from Integrated DNA Technology.

siYAP:

Sense: rGrGrU rCrArG rArGrA rUrArC rUrUrC rUrUrA rArArU rCrAC A

Antisense: rUrGrU rGrArU rUrUrA rArGrA rArGrU rArUrC rUrCrU rGrArC rCrArG

siTAZ:

Sense: rGrCrU rGrCrU rUrCrU rGrGrA rCrCrA rArGrU rArCrA rUrGA A

Antisense: rUrUrC rArUrG rUrArC rUrUrG rGrUrC rCrArG rArArG rCrArG rCrUrG

Negative siRNA:

Sense: rCrGrU rUrArA rUrCrG rCrGrU rArUrA rArUrA rCrGrC rGrUA T

Antisense: rArUrA rCrGrC rGrUrA rUrUrA rUrArC rGrCrG rArUrU rArArC rGrArC

### 4.2 Lipopeptide Synthesis and Purification

CD44-targeting peptide AKPC (Ac-KPSSPPEEGGGKKKGK-PEG4-Cho) was synthesized by conjugating the A6 peptide (KPSSPPEE) with extra GGGKKKGK to a PEG linker and cholesterol. For this, we applied the automatic CEM peptide synthesizer to synthesize the peptide using F-moc chemistry and the standard solid-phase peptide synthesis protocol on a 250 μmol scale, as described previously.^122^ After F-moc deprotection, N_3_-(ethylene glycol)_4_-COOH(N_3_–PEG_4_-COOH) was coupled to both peptides on the resin. This was followed by azide reduction, and cholesteryl-4-amino-4-oxobutanoic acid was coupled to the PEG linker to yield the final product. The control lipopeptide cholesterol-PEG4-scramble A6 (Cho-PEG4-SPEKPEPS-NH2) was synthesized by following the same method. The final products were purified by HPLC using a C18 column, with the confirmation of molecular weight by LC-MS (Figure S1).

### 4.3 Lipid Nanoparticles Formulation

Lipids and lipopeptides (AKPC) from stock solutions were combined at the desired molar ratios, and solvents were evaporated under a nitrogen flow to remove the solvents. The lipid film was dissolved in absolute ethanol and used for the assembly. A solution of siRNA was made by diluting siRNA in 50 mM citrate buffer (pH = 4, RNase-free H_2_O). The solutions were loaded into two separate syringes and connected to a T-junction microfluidic mixer. The solutions were mixed in a 3:1 flow ratio of siRNA:lipids (1.5 mL/min for the siRNA solution, 0.5 mL/min for the lipids solution, N/P ratio was 6:1). After mixing, the solution was directly loaded into a 20 k MWCO dialysis cassette (Slide-A-Lyzer, Thermo Scientific) and dialyzed against 1× PBS overnight. After overnight dialysis, RNA encapsulation efficiency was determined by the Quant-iT RiboGreen RNA Assay Kit, as previously described.^63^ After subtracting the blank measurement, the encapsulation efficiency (in percentage) was calculated as (1 – (nonlysed LNPs/lysed LNPs)) × 100. For cellular binding tests and *in vivo* targeting tests, negative siRNA was encapsulated inside LNPs; 0.5 mol % of DiD was added with the other lipids to form LNPs for confocal imaging and 0.2 mol % of DiD was added with the other lipids to form LNPs for zebrafish imaging. For functional siRNA tests, Yap/TAZ siRNA was encapsulated inside LNPs with a ratio of w/w = 1:2.

### 4.4 Biophysical Characterization

The size and zeta potential of LNPs were measured using a Malvern Nano ZS. The morphology of LNPs was analyzed by cryogenic transmission electron microscopy (cryo-EM). Vitrification of concentrated LNPs (lipids ∼10 mM) was performed using a Leica EM GP operating at 21 °C and 95% relative humidity (RH). Sample suspensions were placed on a glow-discharged 100 μm lacey carbon film supported by 200 mesh copper grids (Electron Microscopy Sciences). Optimal results were achieved using a 60 s preblot and a 1 s blot time. After vitrification, sample grids were maintained below −170 °C, and imaging was performed on a Tecnai T12 (Thermo Fisher) with a biotwin lens and LaB6 filament operating at 120 keV, equipped with an Eagle 4 × 4 K CCD camera (Thermo Fisher). Images were acquired at a nominal underfocus of −2 to −3 μm (49,000× magnification) with an electron dose of ∼2000 e/nm^2^.

### 4.5 Cell Culture and Lentivirus Transfection

HCC38 and MDA-MB-231 cell lines were cultured in Dulbecco′s Modified Eagle′s Medium (DMEM) supplemented with 10% heat-inactivated fetal bovine serum, 1% penicillin/streptomycin (Gibco), and 1% GlutaMAX. To generate HCC38 and MDA-MB-231 cells with red fluorescence and Flip:EGFP/mCherry, we stably transfected the cells with the pLenti-CMV-mCherry and pLenti-Flip:GFP-Caspase 3-T2A-mCherry lentivirus. Fresh medium containing puromycin (2 μg/mL) or blasticidin S (15 μg/mL) (Gibco) was used to select transduced cells with mCherry and Flip:GFP/mCherry expression. Cells were incubated at 37 °C with 5% CO_2_.

### 4.6 Western Blot Analysis

CD44 expressions were analyzed by Western blot as described previously.^123^ Different tumor cells were lysed by lysis buffer, and the lysis sample was subjected to SDS-PAGE to separate the extracted proteins . The blot incubated with a primary mouse monoclonal antibody to human CD44 or β-actin (1:1000, Abcam, ab254530 and Abcam, ab8226) overnight at 4 °C. Then incubated with a secondary antibody (1:2000, horseradish peroxidase-labeled antimouse IgG, Cell Signaling Technology). An Enhanced Chemiluminescence Substrate kit (PerkinElmer) was used to detect the bands and visualized with a ChemiDoC XRS+ System (Bio-Rad).

### 4.7 Cellular Uptake

HCC38 and MDA-MB-231 cells were seeded in a 96-well plate at a density of 2 × 10^4^ cells/well, incubated at 37 °C in 5% CO_2_ the day before, and after 18 h, the cells were added with LNPs (2 μg/mL, 0.5 mol % DiD) and incubated at 4 °C for 30 min. Then, cells were digested, washed, and followed with flow cytometry measurements. For the confocal microscopy measurements, HCC38 and MDA-MB-231 cells were seeded on an 8-well confocal slide at a density of 5 × 10^4^ cells/well and incubated at 37 °C in 5% CO_2_ the day before. Then, Hoechst 33342 (5 μM) was added and incubated for 2 h at 37 °C in 5% CO_2_. LNPs (2 μg/mL siRNA, 0.5 mol % DiD) were incubated for different times and conditions before confocal microscopy imaging.

### 4.8 Zebrafish Xenograft Model of Tumor Cells

The Tg (*fli1*:EGFP)^y1Tg^ Casper transgenic zebrafish were maintained by standard protocols (http://ZFIN.org, in the public domain) and handled in compliance with Dutch animal welfare regulations.^79,124^ The adult zebrafish were placed in a tank with a steep net 1 day in advance, and the zebrafish were naturally fertilized and laid eggs before the light period. Eggs were collected and incubated in egg water (60 μg/mL Instant Ocean sea salts) at 28.5 °C for 2 days. Tumor cells were harvested, centrifuged at 1000 rpm for 5 min, and the pellet which contained the tumor cells was collected, washed with PBS-EDTA, and diluted to 200 cells/nL in 2% polyvinylpyrrolidone-40 (PVP-40; Calbiochem, San Diego, CA, USA). Before injection, embryos were anesthetized with 0.01% tricaine (Sigma-Aldrich Corp., Zwijndrecht, The Netherlands) and placed in a Petri dish with 1% agarose. Resuspended tumor cells were injected via glass capillary needles into the hindbrain via the otic vesicle or Duct of Cuvier of zebrafish embryos at 2 days postfertilization (dpf) at an injection volume of approximately 300–500 cells per fish.

### 4.9 RT-PCR of *In Vitro*

The cells were digested with trypsin and counted before being collected. If the number of cells was between 106 and 107, 500 μL of TRIzol (15596026, Invitrogen) was added for cell lysis. Cell RNA was extracted according to the company’s reagent instructions. The concentration of the extracted RNA was calculated, and the RNA was reverse-transcribed into cDNA using an iScript Reverse Transcription Supermix kit (1708841, Bio-Rad). The intracellular expression of different genes was detected by real-time PCR (Universal SYBR Green Supermix, 1725270, Bio-Rad). The following primers were used to detect genes:

YAP-Forward: TATCAATCCCAGCACAG;

Reverse: GGAATGGCTTCAAGGTAG.

TAZForward: TGGACCAAGTACATGAACCACC;

Reverse: CTGGTGATTGGACACGGTGA.

AMOTL2-Forward: ATTGAGAAGCTGGAAAGCGA;

Reverse: GGTTGAAGTCTTGCAGCCTC.

CTGF-Forward: CAAGGGCCTCTTCTGTGACT;

Reverse: ACGTGCACTGGTACTTGCAG.

CYR61-Forward: CAGCTGACCAGGACTGTGAA;

Reverse: TGTAGAAGGGAAACGCTGCT.

GAPDH-Forward: AGGGCTGCTTTTAACTCTGGT;

Reverse: CCCCACTTGATTTTGGAGGGA.

### 4.10 *In Vitro* Apoptosis Assay

HCC38 and MDA-MB-231 cells were plated at a density of 1 × 10^5^ cells in a 6-well plate. After overnight adherence, cells were treated with LNPs (siRNA, 2 μg/mL) for 4 h. After 48 h of culturing, cells were trypsinized, washed with PBS, and labeled with annexin V-FITC and propidium iodide (PI). Apoptosis was evaluated by flow cytometry, and data were analyzed using CytExpert Software.

### 4.11 Cell Viability Assay

HCC38 and MDA-MB-231 were seeded in 96-well plates at a density of 1 × 10^4^ cells per well. After overnight adherence, the cells were treated with the same volume of PBS and LNPs (siRNA, 2 μg/mL) for 4 h. After 48 h of culturing, cell proliferation reagent WST-1 solution (10 μL, Sigma-Aldrich) was added to the medium (100 μL), and the cells were incubated for an another 4 h at 37 °C. The absorbance at 450 nm was measured at room temperature using a Tecan Infinite M1000. Cell viability was normalized to a control (blank cells), which was set at 100% cell survival.

### 4.12 Colony Formation Assay

HCC38 and MDA-MB-231 cells were seeded into 12-well plates, and 2000 cells were grown in each well. After incubation for 24 h, the same volume of PBS and LNPs (siRNA, 2 μg/mL) was added to the cells and incubated for 4 h. The supernatant was removed, and the cells were the cells were cultured in a fresh culture medium for 12 days at 37 °C. The drug was added again in the same manner on days 4 and 8, respectively. On day 12, the 12-well plate with the cells was incubated on ice for 10 min. After that, the medium was removed, and the cells were washed twice with 1 mL of PBS. 1 mL of ice-cold 100% methanol was used to fix the cells. Finally, the cells were moved off the ice to room temperature and stained with 1% crystal violet for 20 min at room temperature. The colonies were imaged by a GelDoc Go imaging system (Bio-Rad).

### 4.13 Tumor Spheroids Growth Kinetics After LNP Treatments

HCC38 and MDA-MB-231 cells stably expressing Flip:EGFP/mCherry were grown in U-bottom ultralow attachment 96-well plates with 5000 cells per well. After 48 h, we monitored the tumor spheroids using a stereo microscope, recording the fluorescence intensity of Flip:EGFP and mCherry, as well as the volume change of the spheroids. Continuous recordings were made for 12 days to calculate the growth kinetics of the tumor spheres. Three consecutive treatments (siRNA, 2 μg/mL, 4 h) were performed starting on days 0, 4, and 8. Then, the medium was carefully removed from the wells, and fresh medium was added for culturing. The ratio of the mean green fluorescence intensity to the mean red fluorescence intensity in each well was used as quantitative data for cell death. ImageJ was used for image analysis and data calculation.

### 4.14 *In Vivo* Antitumor Evaluation

Prepare zebrafish at 2 dpf and tumor cells with a concentration of 2 × 10^5^ cells/μL as described above. 300–500 cells were implanted in the DoC or hindbrain of 2 dpf zebrafish. One hour later, a 1 nL volume of LNPs (60 pg siRNA) was injected into the dorsal aorta of zebrafish embryos. The injected zebrafish were kept in fresh egg water at 34 °C and after waiting for 18 h, the embryos were anesthetized again using egg water containing 0.01% tricaine. The same dose of LNPs was administered to the zebrafish by IV. Then, place the embryos in glass-bottom Petri dishes and cover them with 1% low-melting agarose containing 0.003% tricaine. Images were acquired using a Leica SP8 confocal microscope and stereo microscopy. Remove the zebrafish from the low-melting agarose. After further cultivation in a 34 °C incubator until 8 dpf, the zebrafish were anesthetized, and tumor growth was recorded using confocal microscopy and stereo microscopy. The data were analyzed by ImageJ.

### 4.15 RT-PCR of Zebrafish Sample

#### 4.15.1 RNA isolation from zebrafish

Zebrafish transplanted with tumor cells were injected with different LNPs and cultured until 4 dpf. Zebrafish were anesthetized by adding tricaine to egg water, and the tails of the fish with tumor cells were cut off on agarose and collected in Trizol (Sigma). RNA isolation from cells: Remove growth media, add 0.3–0.4 mL of Trizol reagent per 1 × 10^5^–10^7^ cells. Whole RNA was extracted using the RNeasy Mini Kit (Qiagen) following the manufacturer’s protocol. After obtaining RNA, the iScript cDNA Synthesis Kit (Bio-Rad) was used for cDNA synthesis, and iQ SYBR Green Supermix (Bio-Rad) was used for qPCR to detect gene expression. Expression of the human housekeeping gene GAPDH was used for normalization. The primers used for qPCR are described as previously (*in the In Vitro* RT-PCR section).

### 4.16 PDX-Derived Organoid *In Vitro* Treatment with LNPs

PDX tumor tissues from LAPC9 PDX were processed for single-cell derivation and organoid culture. Tumors were collected in basal medium (Advanced DMEM F12 serum-free medium [Thermo Fisher Scientific, 12634010] containing 10 mM HEPES [Thermo Fisher Scientific, 15630080], 2 mM GlutaMAX supplement [Thermo Fisher Scientific, 35050061], and 100 μg/mL Primocin [InVivoGen, ant-pm-1]). Tissue dissociation and organoid culture conditions were done as previously described.^108^ Cells were initially seeded in organoid conditions in ultralow attachment plates (Corning, Costar 3474), and after one passage, they were seeded in micropyramid wells SP5D (Kugelmeier Ltd.) at a density of 150 cells/microwell to allow uniform organoid size at the starting point. After 2 days, LNPs were added to LAPC9 organoids (3 duplicate wells per group, siRNA, 2 μg/mL), and the medium was refreshed after 4 h. Organoids were monitored by CQ1 (plate confocal microscope) at different time points (days 2, 4,6, 8, 10, and12).

### 4.17 LNPs Treatment in PCa PDX Mouse Model

Animal experiments were conducted according to the ethical guidelines of Canton Bern, under licenses BE 68/20 and 71/23. For the LAPC9 PDX *in vivo* experiment, tumor tissues were implanted subcutaneously in male immunodeficient CB17-SCID, hormonally intact mice under anesthesia (Domitor 0.5 mg/kg, Dormicum 5 mg/kg, Fentanyl 0.05 mg/kg). Two tumors were implanted per mouse in the scapular region. The tumor cells were priorly stably transduced with fluorescent and bioluminescent reporter (LAPC9-copGFP-CBR, as reported,^109^ which allow for intravital imaging and tumor growth assessment. One week post-tumor implantation, tumor bioluminescence measurements were taken using IVIS-CT imaging, and, together with body weight measurements, were used to randomize into treatment groups. LNP treatment at 1.0 mg/kg was done via 3× injections every 2 days for a total of 1 week, and each tumor was injected with 10 μg of siRNA. Subcutaneous injections of LNPs dissolved in PBS were performed (10 μg per tumor, 25 G needle). Tumor growth was monitored for 2 more weeks post-treatment by IVIS-CT. d-luciferin (150 mg/kg) was injected into the mice subcutaneously, and imaging took place after 20 min. At 4 weeks postimplantation, LAPC9 tumors were collected, and tumor size was measured by caliper.

### 4.18 Statistical Analysis

The experiments of LNPs targeting tumor cells in zebrafish were repeated twice. In the tumor suppression experiments of the zebrafish model, 30 zebrafish were used for statistical analysis in each group. For all repeated experiments, freshly prepared LNPs were used. GraphPad Prism 6 software was used for statistical analysis. The results are presented as mean ± SD. Two-way ANOVA was used to analyze more than two groups, followed by Bonferroni posttest. (****, *p* < 0.0001; ***, *p* < 0.001; **, *p* < 0.01; *, *p* < 0.05; ns, no significant difference).

## Supporting Information Available

LC-MS spectrum of lipopeptide AKPC; cellular binding of LNPs; biodistribution of LNPs by confocal microscopy; *in vivo* tumor-targeting of LNPs on MDA-MB-231 cells in tail of zebrafish; tumor targeting of LNPs to HCC38 cells in hindbrain of zebrafish; *in vivo* tumor-targeting of LNPs on MDA-MB-231 cells in hindbrain of zebrafish; single-cell image of breast cancer cells isolated from zebrafish; *in vitro* antitumor effect of LNPs encapsulating YAP/TAZ-siRNA on MDA-MB-231 cells; evaluation of the antitumor effect of LNPs encapsulating YAP/TAZ-siRNA on tumor spheroids of HCC38; tumor xenograft and growth in zebrafish; tolerance evaluation of LNPs encapsulating YAP/TAZ-siRNA in zebrafish; HCC38 tumor implantation in one-day postinjection zebrafish; therapeutic antitumor effect of LNPs encapsulating YAP/TAZ-siRNA *in vivo* of MDA-MB-231; therapeutic antitumor effect of LNPs encapsulating YAP/TAZ-siRNA *in vivo*; body weight of mice per group during the LNPs encapsulating YAP/TAZ-siRNA treatment experiment timeline (PDF)
High-resolution single-cell imaging revealed that HCC38 and MDA-MB-231 cells exhibited greater internalization of AKPC-siYT compared to MC3-siYT(MP4)
