# Supplementary material for: Peptide-Modified Lipid Nanoparticles Boost the Antitumor Efficacy of RNA Therapeutics
Source: ACS Nano. 2025 Apr 3;19(14):13685–704. doi: 10.1021/acsnano.4c14625 (PMC12004924; doi:10.1021/acsnano.4c14625)
Supplement: Supplementary file 1 — nn4c14625_si_001.pdf [file nn4c14625_si_001.pdf]

Supporting information

Peptide-Modified Lipid Nanoparticles Boost the Anti-tumor Efficacy of RNA Therapeutics

Gangyin Zhao,[1,6#] Ye Zeng,[2,5#] Wanli Cheng,[3] Sofia Karkampouna,[3,4] Panagiota Papadopoulou,[2] Bochuan Hu,[2] Shuya Zang,[2] Emma Wezenberg,[2] Gabriel Forn-Cuní,[1] Bruno Lopes Bastos,[1] Marianna Kruithof-de Julio,[4] Alexander Kros,[2*] and B. Ewa Snaar-Jagalska[1*]*

1. Department of Cellular Tumor Biology, Leiden Institute of Biology, Leiden University, Einsteinweg 55, 2333 CC Leiden, The Netherlands
2. Department of Supramolecular & Biomaterials Chemistry, Leiden Institute of Chemistry, Leiden University, Einsteinweg 55, 2333 CC Leiden, The Netherlands
3. Urology Research Laboratory, Department for BioMedical Research, University of Bern, 3010 Bern, Switzerland
4. Department of Urology, Inselspital, Bern University Hospital, University of Bern, 3010 Bern, Switzerland
5. Present address: Department of Bioengineering, University of Pennsylvania, Philadelphia, PA 19104, USA.
6. Shenzhen Institute of Advanced Technology, Chinese Academy of Sciences, Shenzhen, 51800, China.

[#] These authors contributed equally: Gangyin Zhao, Ye Zeng.

*Correspondence:

Ye Zeng, School of Engineering and Applied Science, University of Pennsylvania, Philadelphia,

PA, USA, E-mail: yezeng@seas.upenn.edu

Alexander Kros, Leiden Institute of Chemistry, Leiden University, Einsteinweg 55, 2333 CC Leiden, The Netherlands, E-mail: a.kros@chem.leidenuniv.nl

Ewa Snaar-Jagalska, Leiden Institute of Biology, Leiden University, Einsteinweg 55, 2333 CC Leiden, The Netherlands, E-mail: b.e.snaar-jagalska@biology.leidenuniv.nl

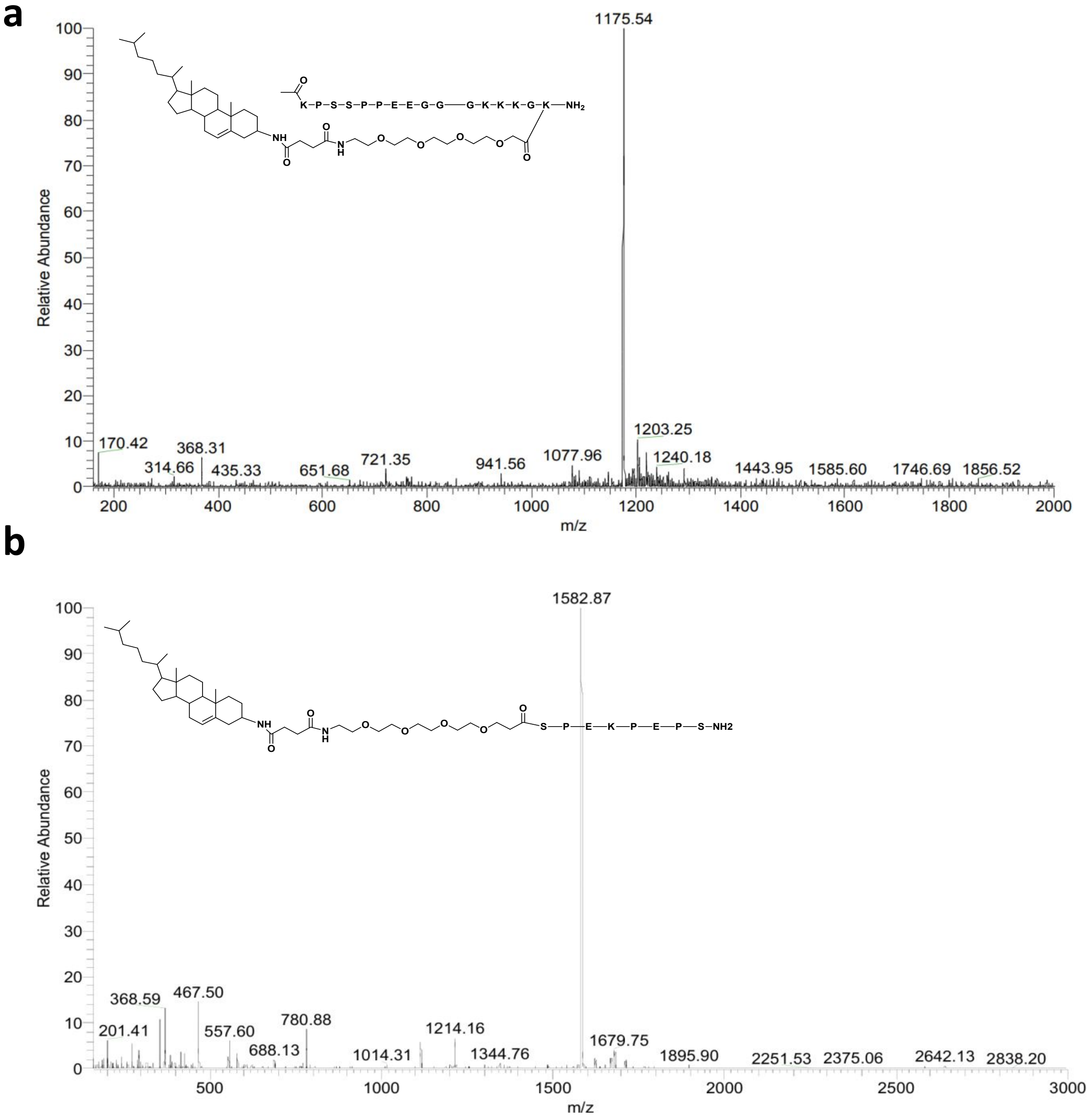

Figure S1. **LC-MS spectrum of CD44 targeting lipopeptide AKPC (a) and control lipopeptide cholesterol-PEG4-ScrambleA6 (b).**

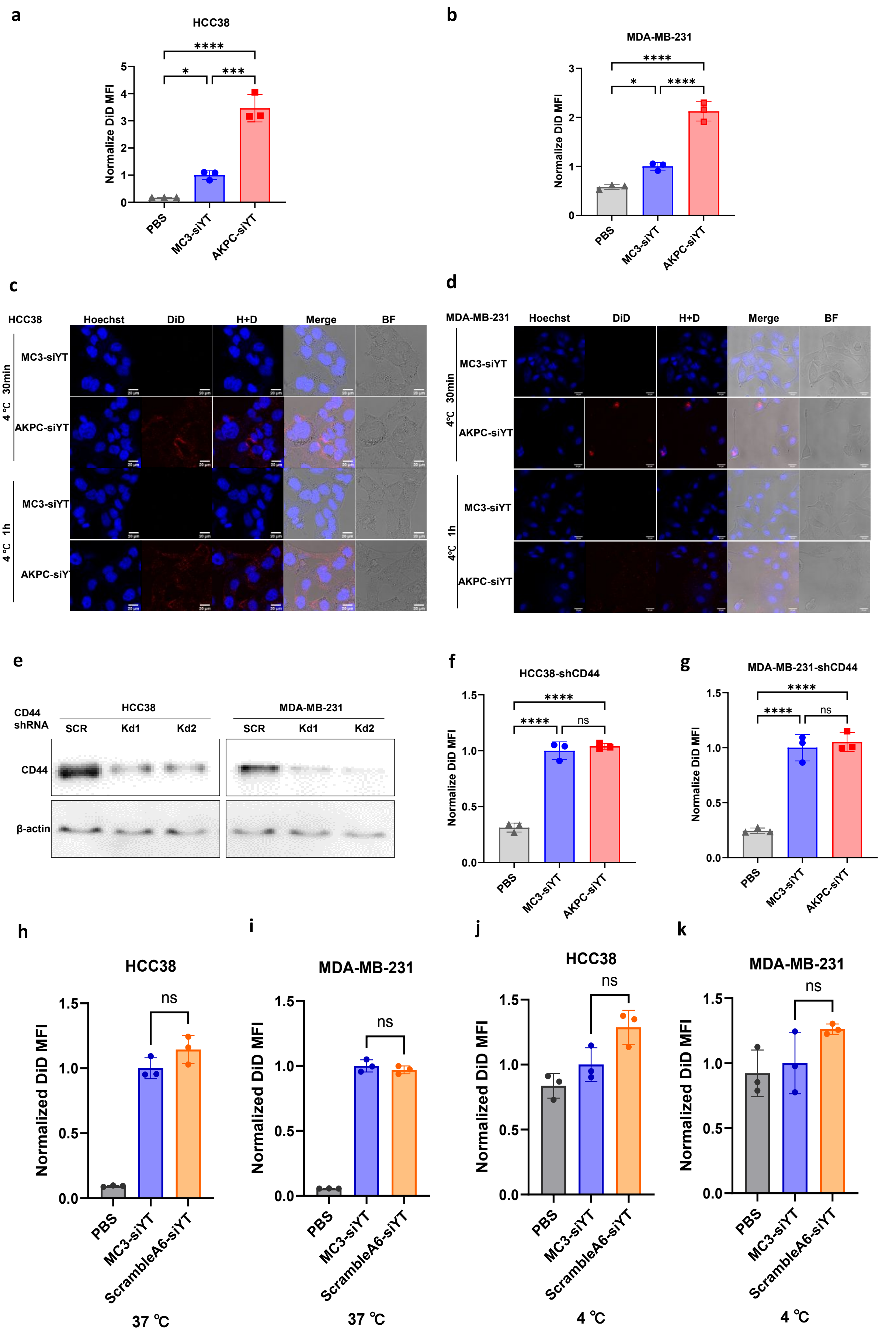

Figure S2. Cellular binding of LNPs. a,b, Cellular internalization efficiency of DiD-labelled LNPs with

HCC38 and MDA-MB-231 cells by FACS, DiD positive percentage indicates the percentage of tumor cells with LNP uptake. **c**,**d**, Confocal microscopic images of cellular internalization of LNPs on HCC38 and MDA-MB-231 cells at 4 °C after 30 min and 1 hour incubation. 0.5 mol% DiD was added to the lipids and served as the fluorescent dye. Scale bar represents 20 µm. **e**, Western blot images of CD44 expression in CD44 knockdown breast cancer cell lines, Kd1: Cells stably expressing CD44 shRNA, named as Kd1; Kd2: Cells stably expressing different sequences of CD44 shRNA, named as Kd2. **f**,**g**, Cellular internalization efficiency of LNPs to CD44 knockdown cell lines quantified by flow cytometry. Ordinary one-way ANOVA was used to determine the significance of the comparisons of data IN a, b, d, and e (*P < 0.05; **P < 0.01; ***P < 0.001; ****P < 0.0001). In all panels, error bars represent mean±s.d. (n=3) In all panels, error bars represent mean±s.d. (n=3). **h**-**k**, Cellular internalization efficiency of peptide modified LNPs with HCC38 and MDA-MB-231 cells in 4℃ and 37℃ by FACS, DiD positive percentage indicates the percentage of tumor cells with LNP uptake.

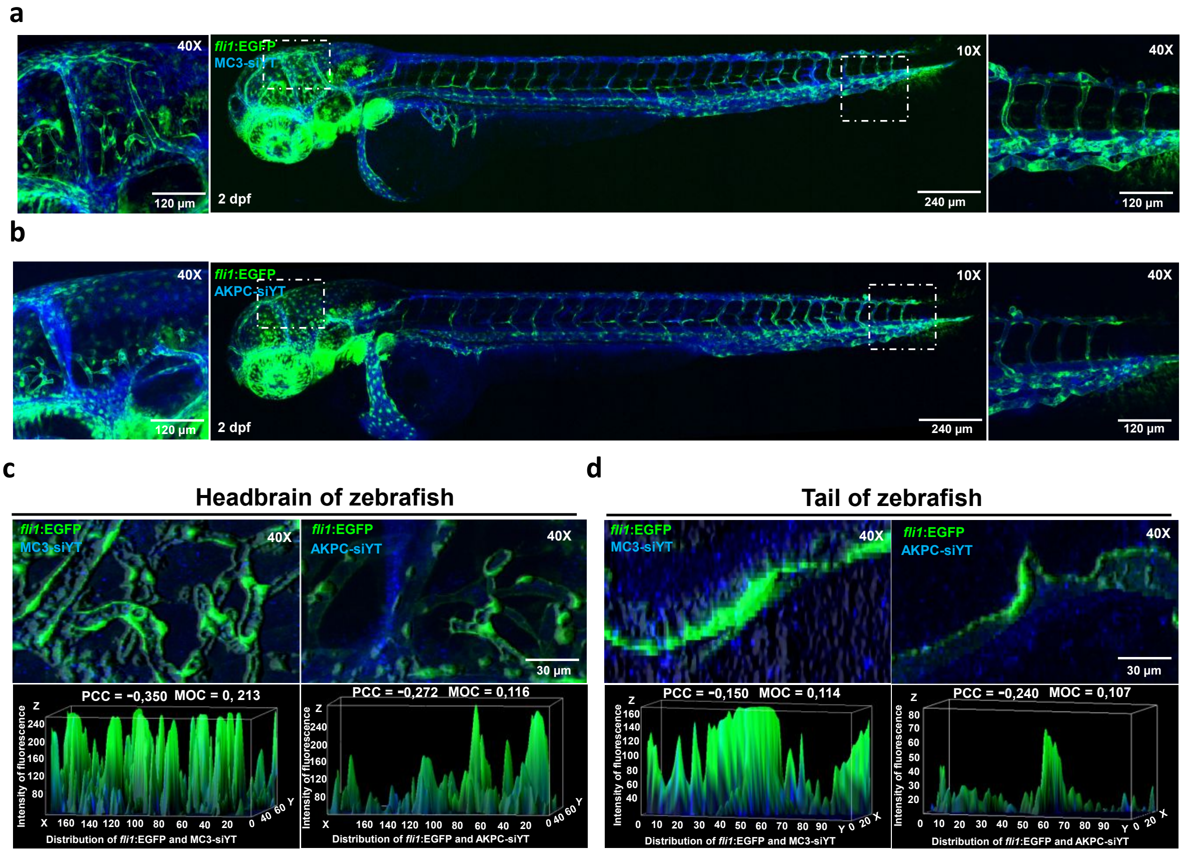

Figure S3. Biodistribution of LNPs by confocal microscopy. **a**, Representative images of biodistribution of LNP (blue) at 4 hpi in the whole zebrafish (10X) and local distribution image of hindbrain and tail (40X). *fli1*:EGFP/Casper zebrafish (green) was used. (n=5) **b**, Representative images of biodistribution of AKPC-LNP at 4 hpi in the whole zebrafish (10X) and local distribution image of hindbrain and tail (40X) (n=5). Scale bars: 240 µm (whole embryo) and 120 µm (tissue level). **c**,**d**, Local images of the 40X brain and tail were zoomed and co-localization analysis of LNPs and blood vessels was performed by calculating PCC and MOC. The peak map showed the co-distribution of *fli1*:EGFP and LNP, and the peak showed fluorescence intensity.

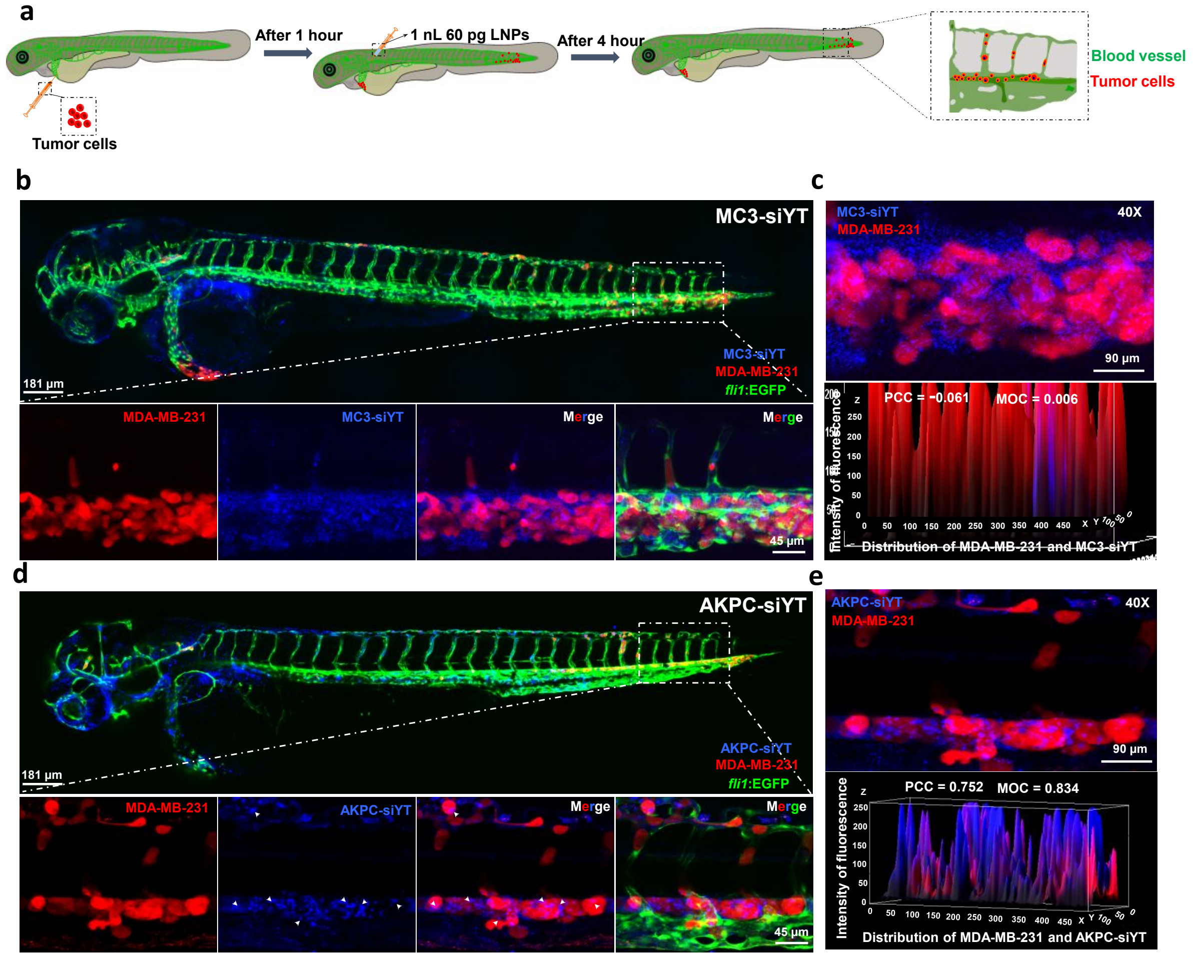

Figure S4. ***In vivo*** **tumor-targeting of LNPs on MDA-MB-231 cells in tail of zebrafish.** **a**, Schematic representation of *in vivo* targeting of breast cancer cells in the CHT region of the tail. **b**,**d**, mCherry marker MDA-MB-231 was implanted into DoC of *fli1*:EGFP/Casper zebrafish. 0.2 mol% DiD was added to the lipids and served as the fluorescent dye. After IV injection of tumor cells and LNPs (60 pg siRNA), confocal imaged the LNPs binding to tumor cells in the circulation of zebrafish. **c**,**e**, Image J calculated the colocalization of MDA-MB-231 and LNPs in the circulation of zebrafish, and calculate PCC and MOC values.

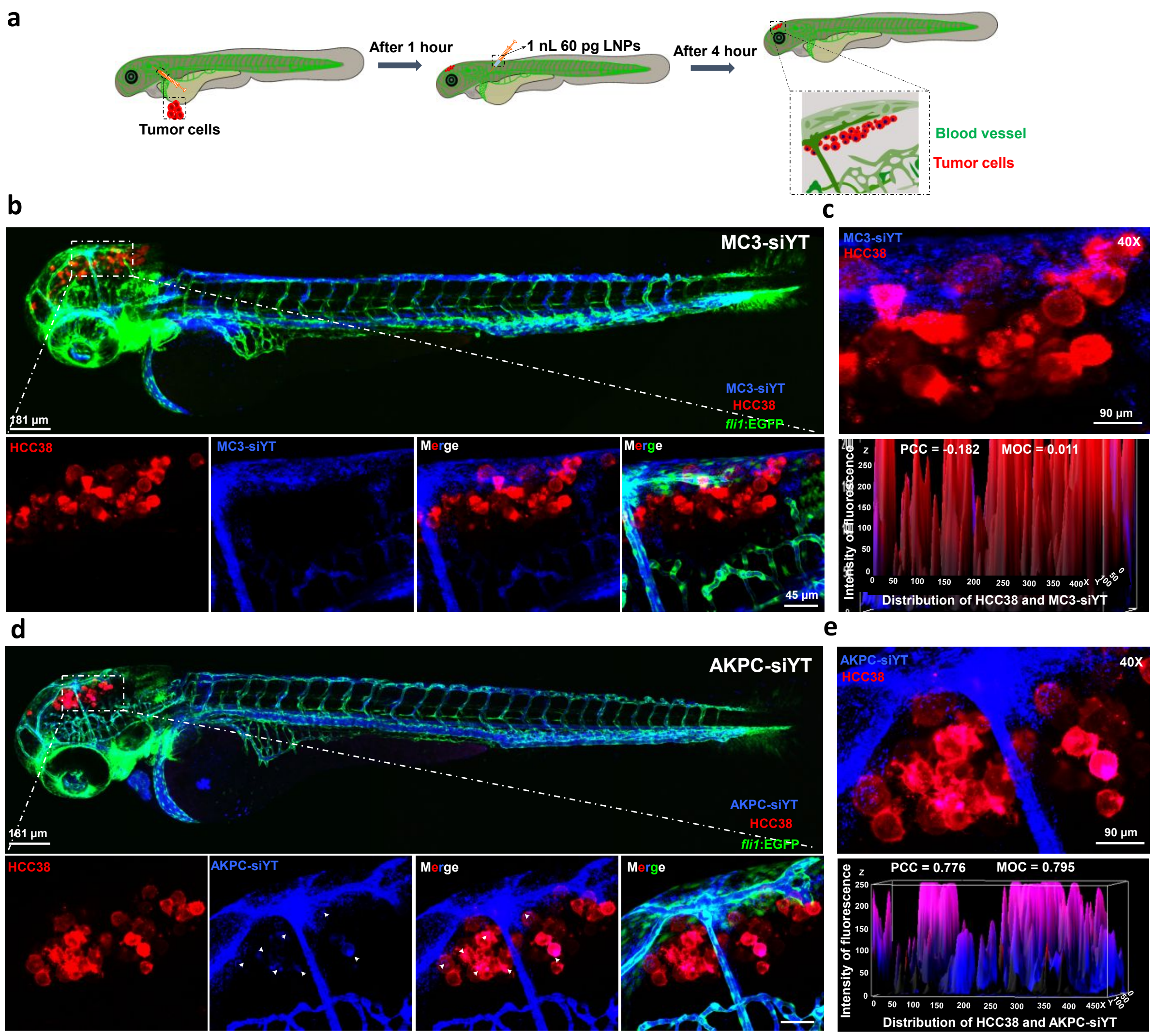

Figure S5. Tumor targeting of LNPs to HCC38 cells in hindbrain of zebrafish. a, Schematic representation of *in vivo* tumor targeting through the hindbrain. **b**,**d**, One hour after HCC38 cells were implanted into the hindbrain of 2 dpf zebrafish, LNPs were injected into the circulation of zebrafish. **c**,**e**, Image J calculated the colocalization of HCC38 and LNPs in the hindbrain of zebrafish in the same way as in the tail. The images were acquired by SP8 confocal microscope. All the data were representative of three independent experiments (each n=5) and analyzed by image J software.

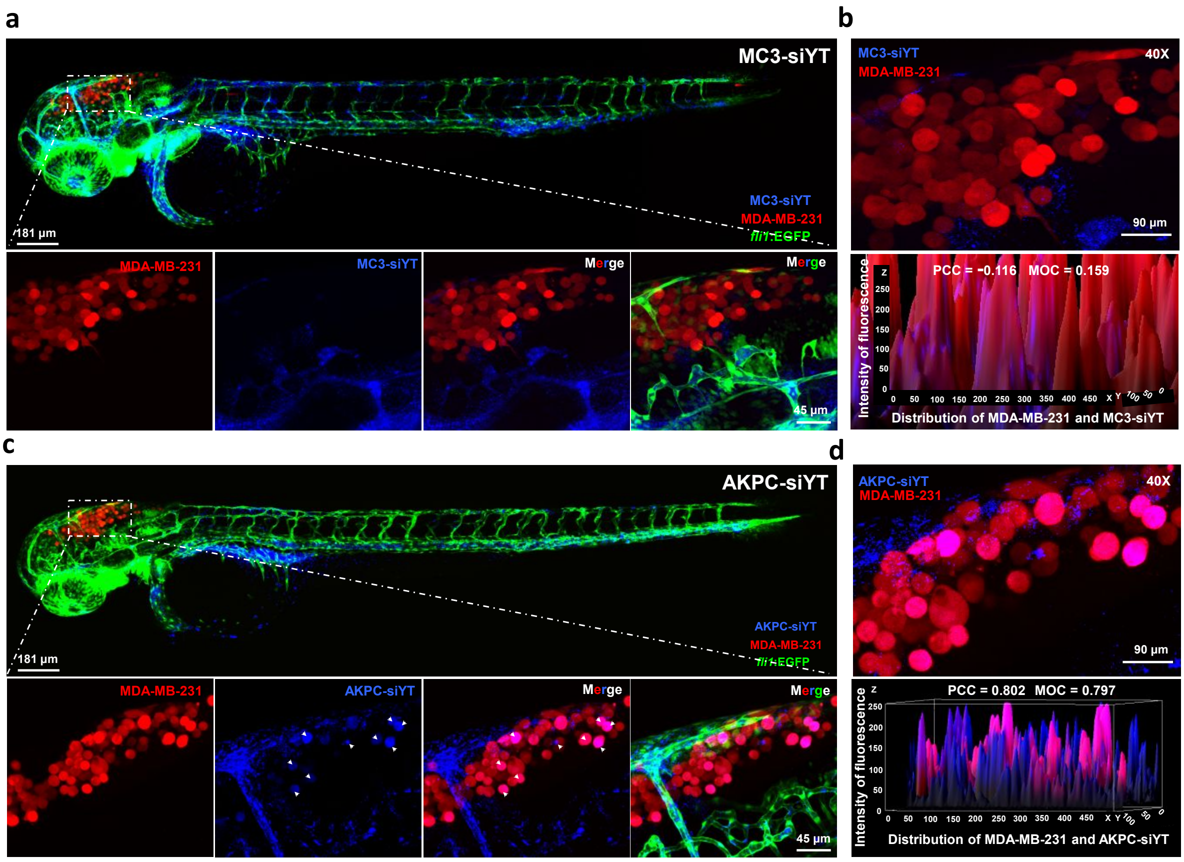

Figure S6. *In vivo* tumor-targeting of LNPs on MDA-MB-231 cells in hindbrain of zebrafish. a,**c**, MDA-MB-231 was injected into the hindbrain of zebrafish, and the IV injection of LNPs after one-hour cells was injected. **b**,**d**, Image J calculated the colocalization of MDA-MB-231 and LNPs as well as PCC and MOC in the hindbrain of zebrafish. Confocal was used to detect LNP binding to tumor cells in the hindbrain. All data were obtained from three independent replicates (each=5), and were analyzed by image J.

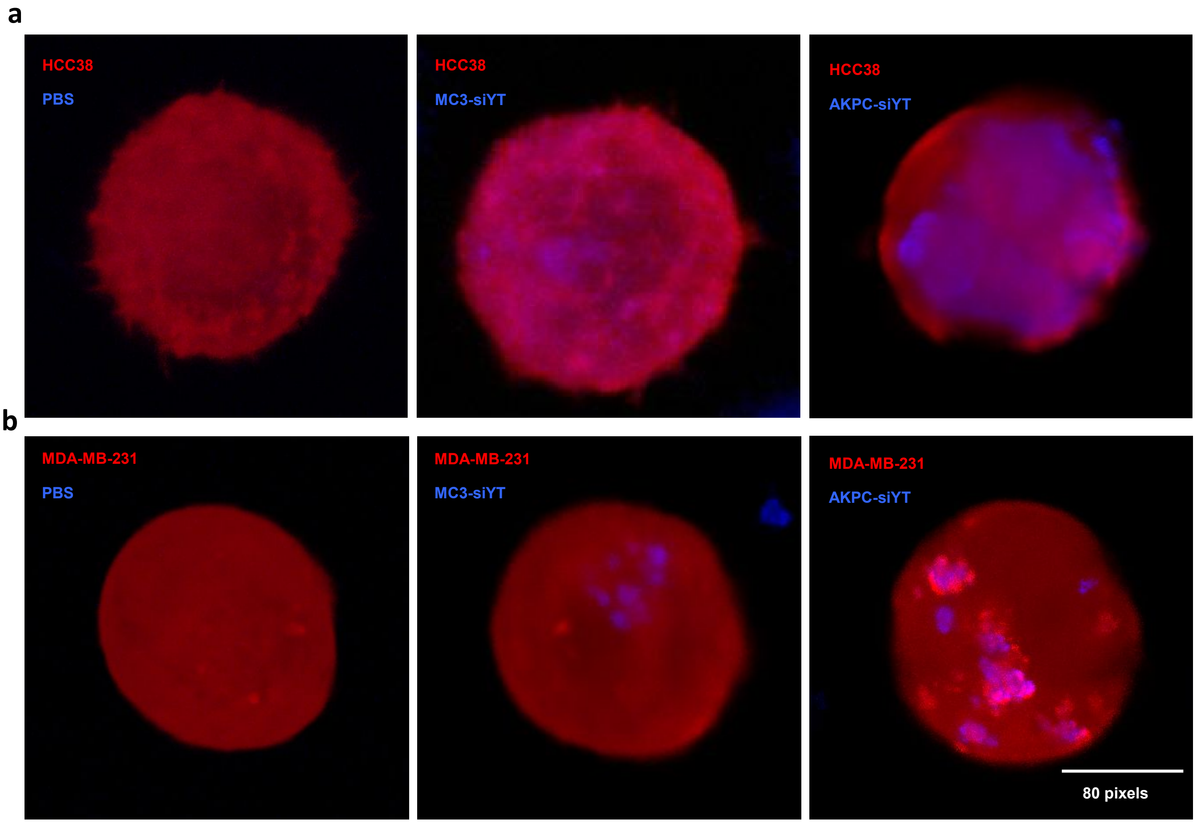

Figure S7. Single-cell image of breast cancer cells isolated from zebrafish. a, High resolution Single-cell image of HCC38 isolated from zebrafish tail took by confocal microscopy (63X). HCC38 is Red and LNPs are blue. **b**, High resolution single-cell image of MDA-MB-231 isolated from zebrafish took by confocal microscopy (63X). MDA-MB-231 is Red and LNPs are blue.

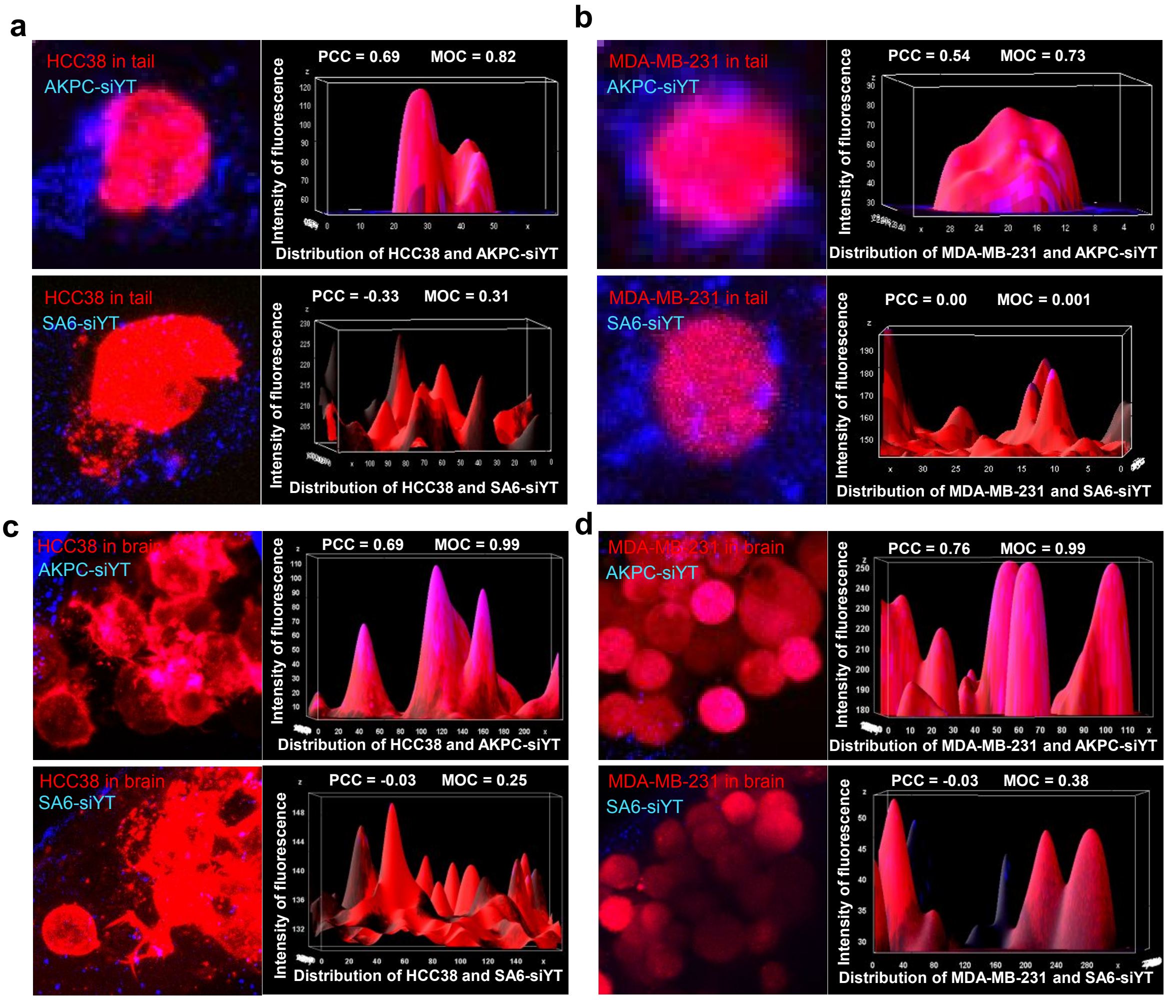

Figure S8. ***In vivo*** **tumor-targeting of SA6-siYT and AKPC on tumor cells in zebrafish. a**,**c**, Tumor cells were injected into the DoC of zebrafish, and the IV injection of AKPC-siYT/SA6-siYT after one-hour cells was injected. Confocal was used to detect LNP binding to tumor cells in the hindbrain. Image J calculated the colocalization of HCC38/MDA-MB-231 and LNPs as well as PCC and MOC in the tail of zebrafish. **b**,**d**, Tumor cells were injected into the hindbrain of zebrafish, and the IV injection of AKPC-siYT/SA6-siYT after one-hour cells was injected. Confocal was used to detect LNP binding to tumor cells in the hindbrain. Image J calculated the colocalization of HCC38/MDA-MB-231 and LNPs as well as PCC and MOC in the hindbrain of zebrafish.

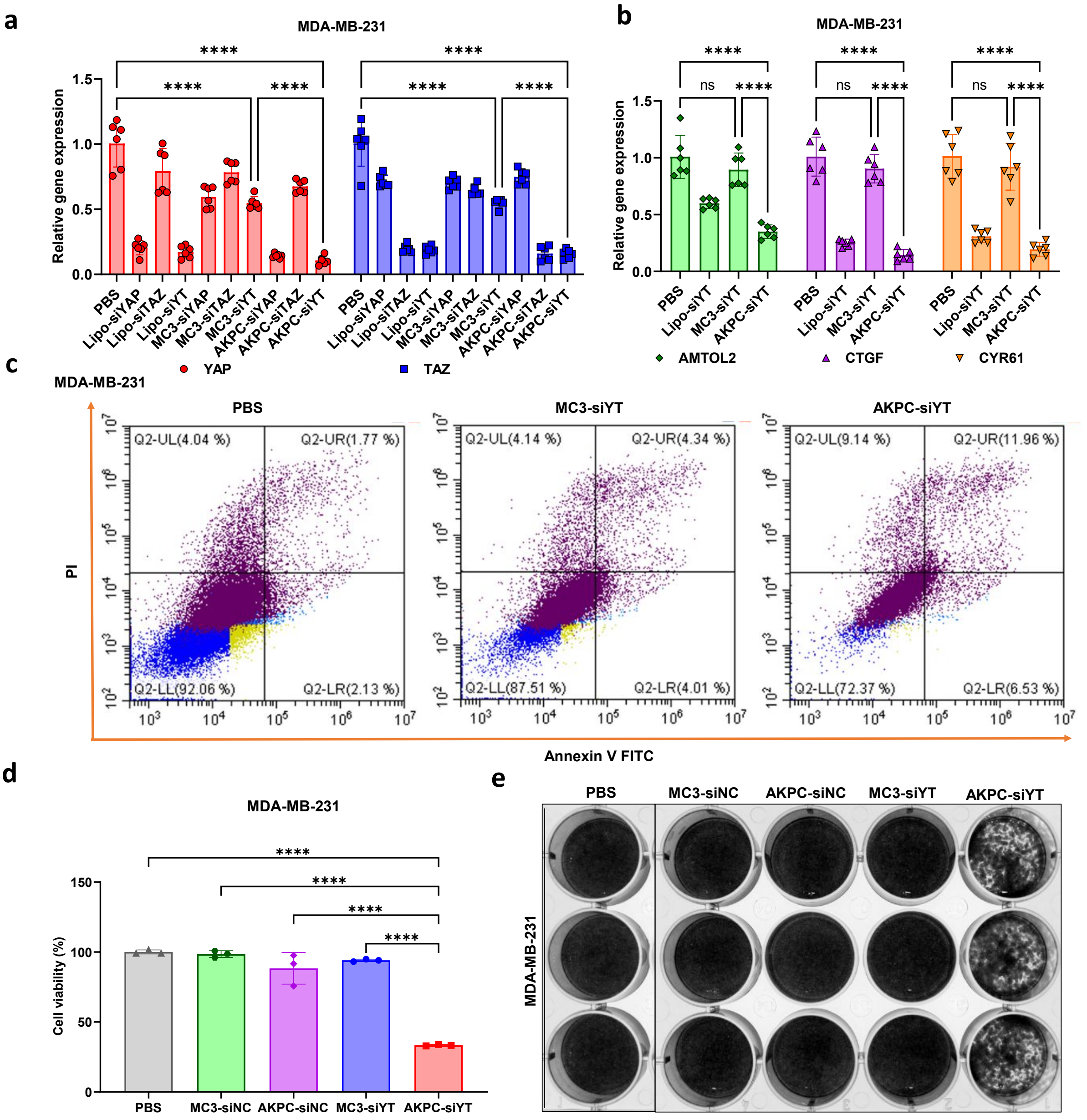

Figure S9. ***In vitro*** **anti-tumor effect of LNPs encapsulating YAP/TAZ-siRNA on MDA-MB-231 cells.** **a**, RT-PCR results after different co-delivery siYAP and siTAZ in MDA-MB-231 cells. **b**, RT-PCR results of the downstream gene after co-delivery of siYAP and siTAZ in MDA-MB-231 cells. Two-way ANOVA was used to determine the significance of the comparisons of data indicated in a and b (*P < 0.05; **P < 0.01; ***P < 0.001; ****P < 0.0001). In all panels, error bars represent mean±s.d. (n=3). **c**, Annexin V/PI staining of MDA-MB-231 cells after treatments of LNPs. **d**, Cell viability measurements by WST-1 in MDA-MB-231 cells after treatments of LNPs. Ordinary one-way ANOVA was used to determine the significance of the comparisons of data (*P < 0.05; **P < 0.01; ***P < 0.001; ****P < 0.0001). In all panels, error bars represent mean±s.d. (n=3). **e**, Cell viability of crystal violet staining in MDA-MB-231 cells after treatments of LNPs.

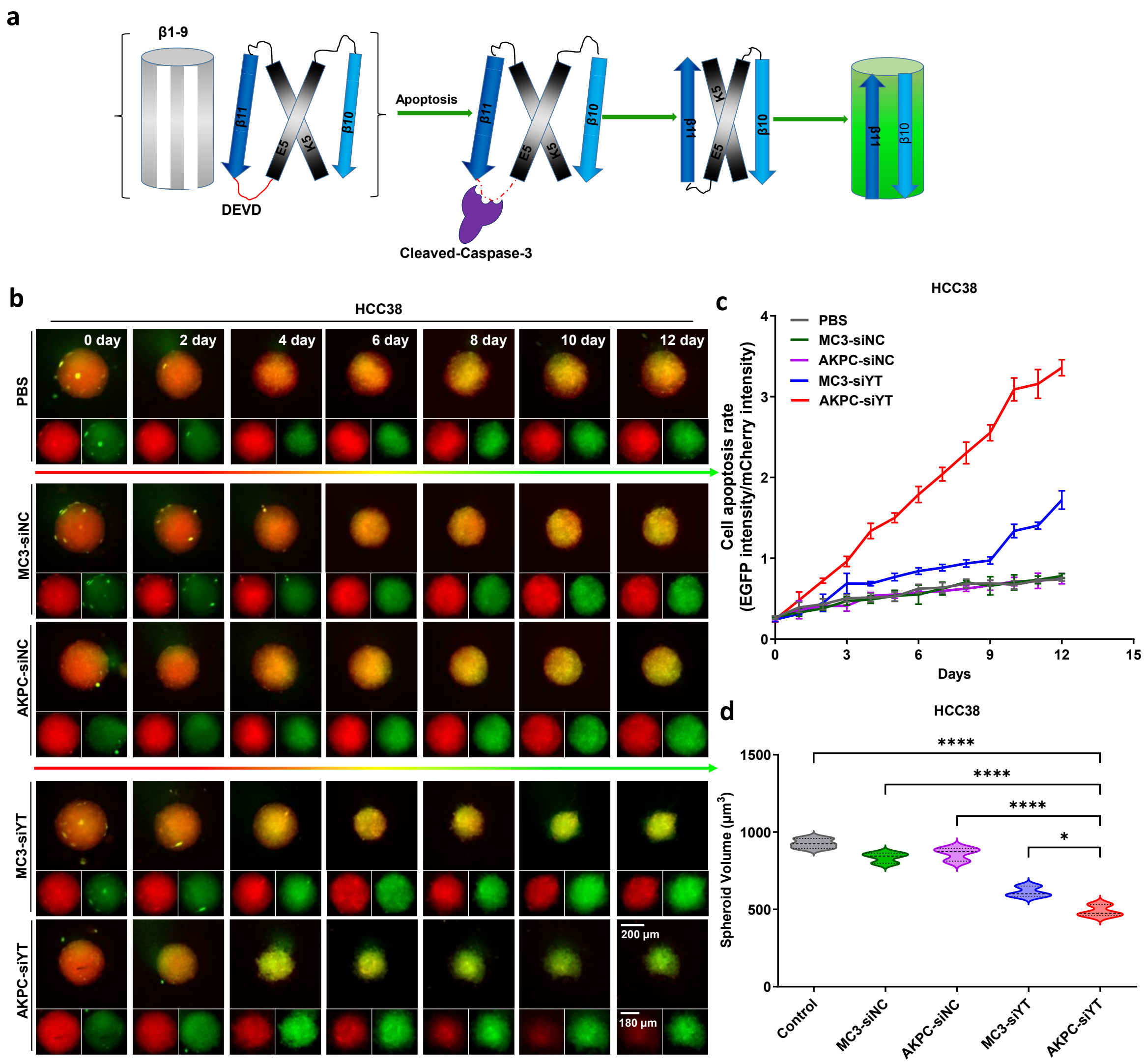

Figure S10. Evaluation of the anti-tumor effect of LNPs encapsulating YAP/TAZ-siRNA on tumor spheroids of HCC38. a, Schematic representation of plasmid containing GFP-T2A-Caspase3-mCherry (cell apoptosis sensor). **b**, HCC38 treated with LNPs was photographed continuously for 12 days by stereo microscope. **c**, The kinetics of cell apoptosis rate from HCC38 spheroid over time after treatments of LNPs. **d**, HCC38 spheroids volume on day 12. Ordinary one-way ANOVA was used to determine the significance of the comparisons of data (*P < 0.05; **P < 0.01; ***P < 0.001; ****P < 0.0001). In all panels, error bars represent mean±s.d. (n=3).

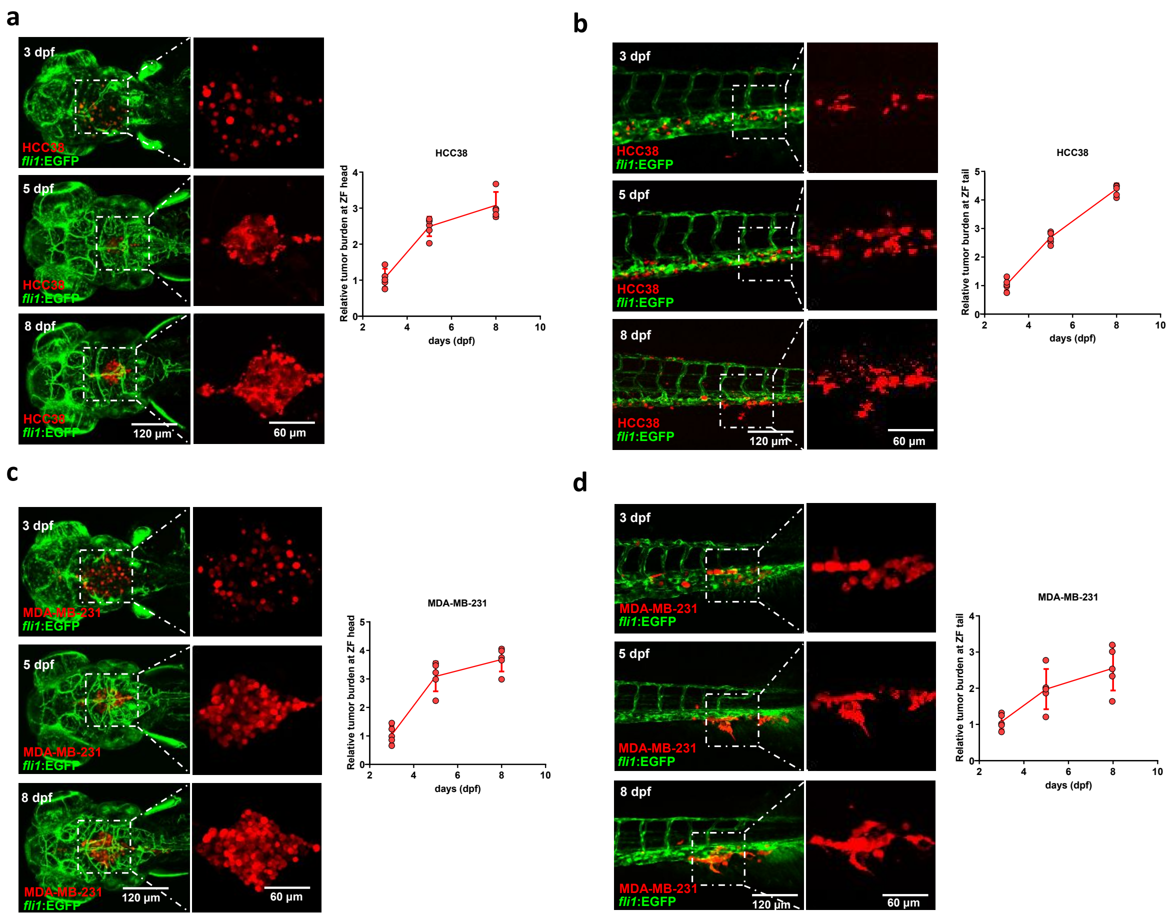

Figure S11. Tumor xenograft and growth in Zebrafish. a,**b**, SP8 confocal microscope imaged the HCC38 (in red) growth in the hindbrain and circulation of zebrafish (blood vessel in green) at 3 dpf, 5 dpf, and 8 dpf. The means of HCC38 in the hindbrain and tail of zebrafish at 3 dpf, 5 dpf, and 8 dpf normalize to the means at 3 dpf (mean ± s.d., n=5). **c**,**d**, Recorded MDA-MB-231 tumor growth at 3 dpf, 5 dpf, and 8 dpf by confocal microscope. The means of MDA-MB-231 in the hindbrain and tail of zebrafish at 3 dpf, 5 dpf, and 8 dpf normalize to the means at 3 dpf (mean ± s.d., n=5). All data were analyzed by image J.

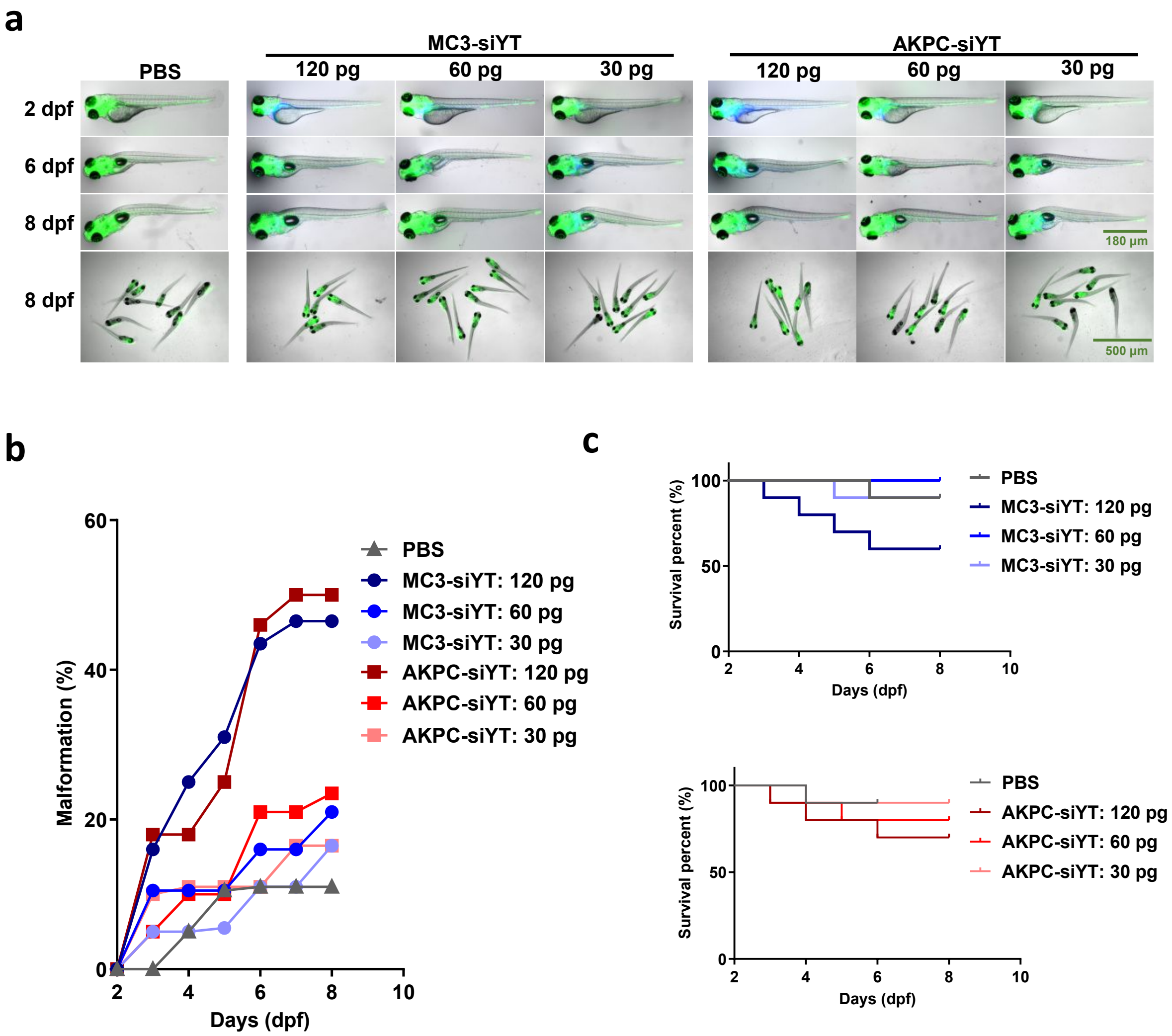

Figure S12. Tolerance evaluation of LNPs encapsulating YAP/TAZ-siRNA in zebrafish. **a**, Zebrafish were photographed by stereo microscope for six days after IV injections of different concentrations of LNPs. **b**, The malformation rate of zebrafish was calculated within six days after LNP injection (mean ± s.d., n=10/group). **c**, Survival curves of zebrafish within six days after LNPs injection (n=10).

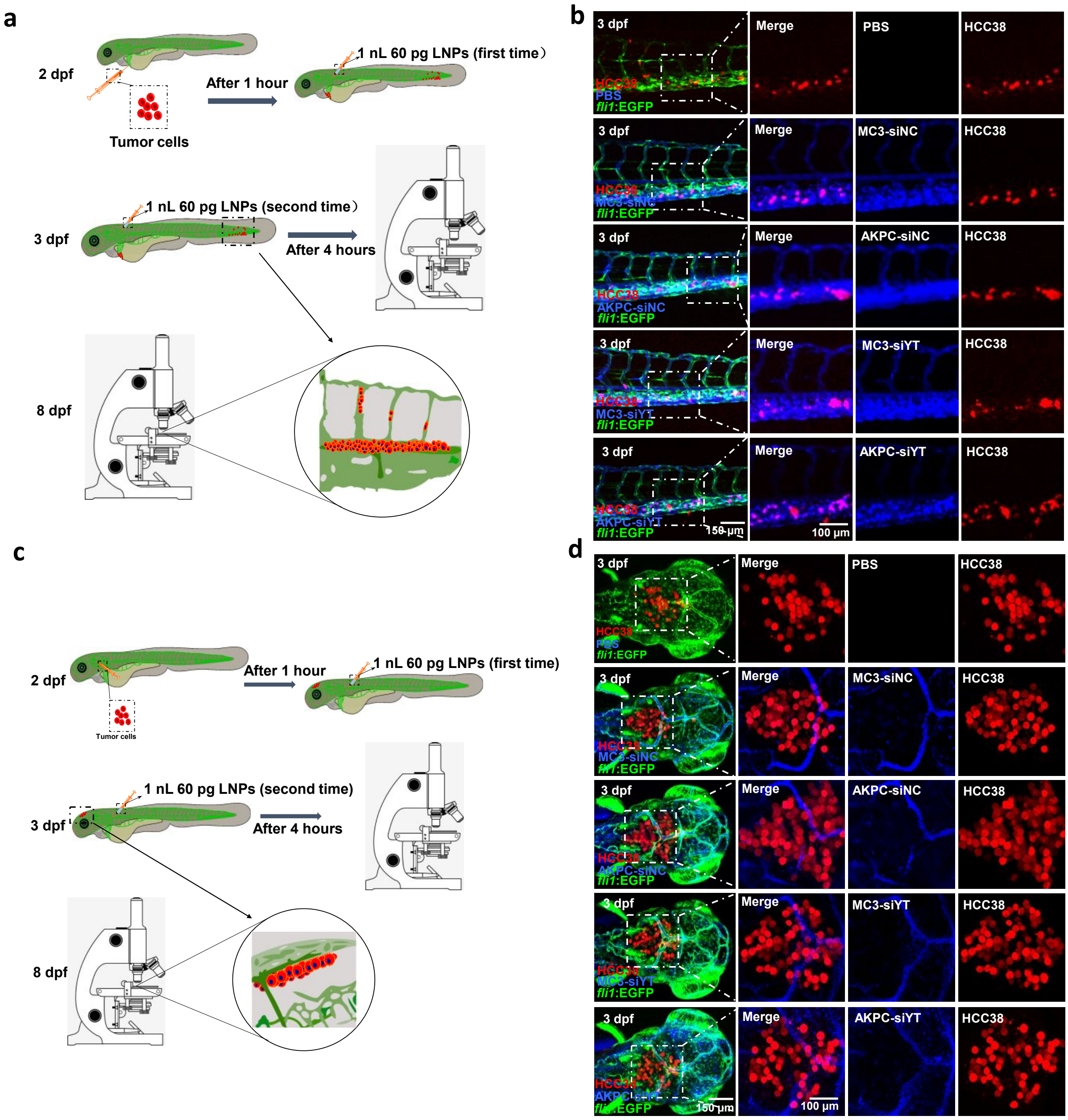

Figure S13. HCC38 tumor implantation in one-day post-injection zebrafish. a,**c**, Schematic representation of LNPs treatments of tumor in tail (DoC injection) and in head (hindbrain injection). **b**, The image of mCherry-HCC38 tumor burden (in Red) with LNPs-siRNA (in blue) in the circulation of Fli: GFP/Casper (in Green) zebrafish at 3 dpf. Zebrafish transplanted with HCC38 were injected with PBS, MC3-siNC, AKPC-siNC, MC3-siYT and AKPC-siYT by IV administration (n=30/group). **d**, The image of mCherry-HCC38 tumor burden (in Red) with LNPs-siRNA (in blue) in the hindbrain of Fli: GFP/Casper (in Green) zebrafish at 3 dpf (n=30/group). All data were analyzed by image J.

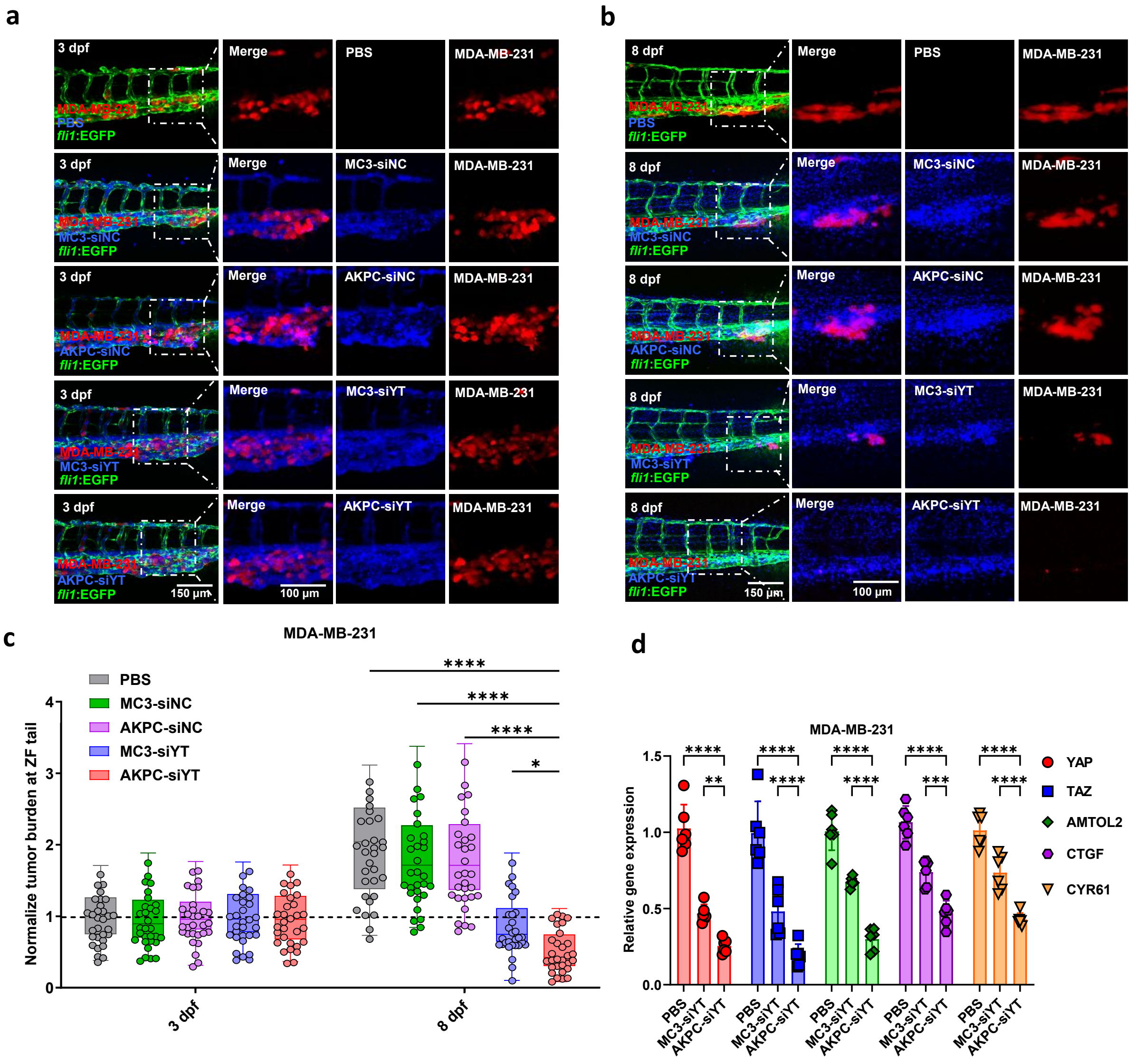

Figure S14. Therapeutic anti-tumor effect of LNPs encapsulating YAP/TAZ-siRNA *in vivo* of MDA-MB-231. a,**b**, The images of MDA-MB-231 implanted in the zebrafish circulation with LNPs-siRNA at 3 dpf and 8 dpf. **c**, Relative fluorescence intensity (The ratio of fluorescence intensity of each group at 8 dpf to that of PBS group at 3 dpf) of MDA-MB-231 tumor burden in zebrafish CHT at 3 dpf and 8 dpf (mean ± s.d., n=30/group). Two-way ANOVA multiple comparisons were used to determine the significance of the comparisons of data (*P < 0.05; **P < 0.01; ***P < 0.001; ****P < 0.0001). In all panels, error bars represent mean±s.d. **d**, RT-PCR results of YAP/TAZ and downstream gene expression in zebrafish after co-delivery of siYAP and siTAZ at 8 dpf. Two-way ANOVA was used to determine the significance of the comparisons of data (*P < 0.05; **P < 0.01; ***P < 0.001; ****P < 0.0001). In all panels, error bars represent mean±s.d. (n=3).

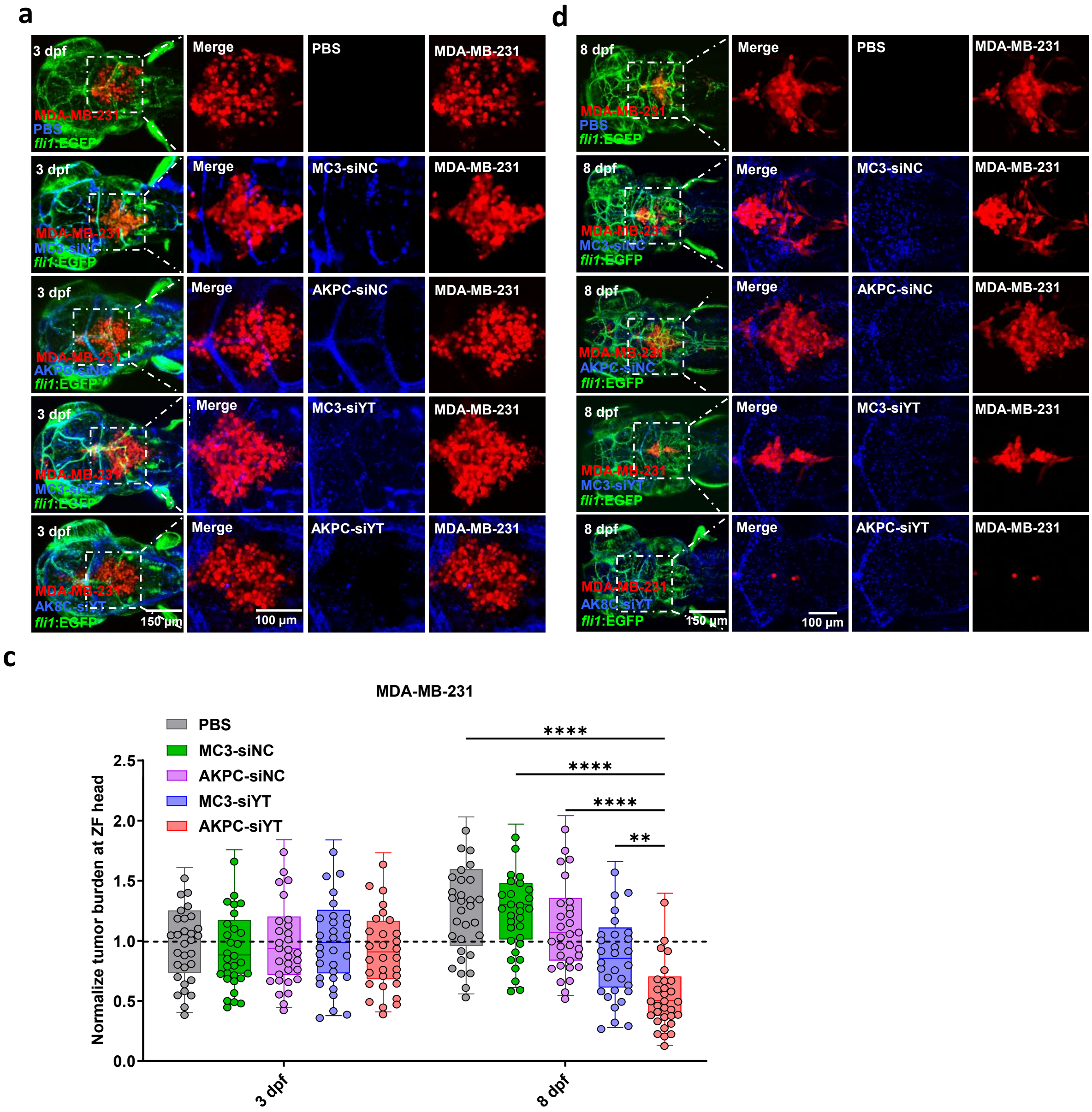

Figure S15. Therapeutic antitumor effect of LNPs encapsulating YAP/TAZ-siRNA in *vivo*. a,b, The image of MDA-MB-231 tumor burden with LNPs-siRNA in the hindbrain at 3 dpf and 8 dpf. **c**, The relative intensity of red fluorescence (the ratio of fluorescence intensity of each group at 8 dpf to that of PBS group at 3 dpf) was used to measure tumor burden at the hindbrain in Zebrafish at 3 dpf and 8 dpf (mean ± s.d., n=30/group). Two-way ANOVA multiple comparisons were used to determine the significance of the comparisons of data (*P < 0.05; **P < 0.01; ***P < 0.001; ****P < 0.0001). In all panels, error bars represent mean±s.d.

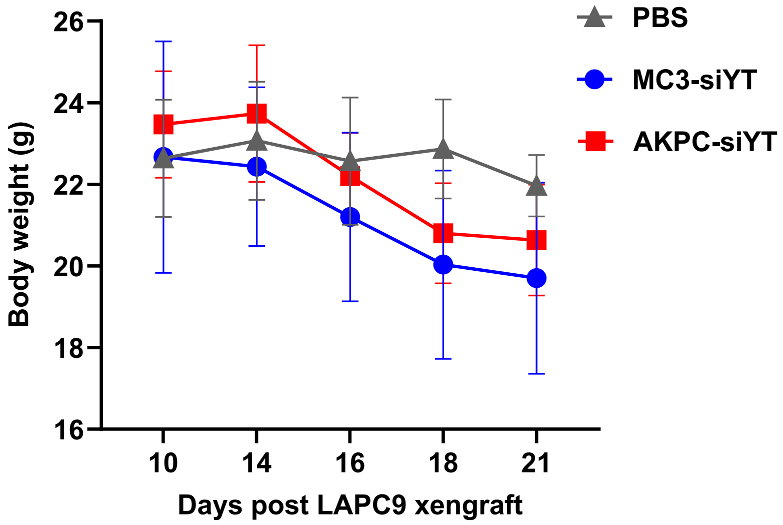

Figure S16. Body weight of mice per group during the LNPs encapsulating YAP/TAZ-siRNA treatment experiment timeline.
